# Current biodiversity status, distribution, and prospects of seaweed in Indonesia: A systematic review

**DOI:** 10.1016/j.heliyon.2024.e31073

**Published:** 2024-05-10

**Authors:** Mohammad Basyuni, Maya Puspita, Rinny Rahmania, Hatim Albasri, Indra Pratama, Dini Purbani, A.A. Aznawi, Alfian Mubaraq, Shofiyah S. Al Mustaniroh, Firman Menne, Yulizar Ihrami Rahmila, Severino G. Salmo III, Arida Susilowati, Siti H. Larekeng, Erwin Ardli, Tadashi Kajita

**Affiliations:** a Center of Excellence for Mangrove, Universitas Sumatera Utara, Medan, 20155, Indonesia; b Department of Forestry, Faculty of Forestry, Universitas Sumatera Utara, Medan, 20155, Indonesia; c Asosiasi Rumput Laut Indonesia, Jakarta, Indonesia; d Research Center for Ecology and Ethnobiology, National Research and Innovation Agency, Cibinong, 16911, Indonesia; e Research Center for Fisheries, National Research and Innovation Agency, Cibinong, 16911, Indonesia; f Research Center for Conservation of Marine Resources and Inland Waters, Cibinong, 16911, Indonesia; g Department of Accounting, Faculty of Economics and Business, Universitas Bosowa, Makassar, Indonesia; h Institute of Biology, College of Science, University of the Philippines Diliman, Quezon City, Philippines; i Biodiversity Research Group, Faculty of Forestry, Hasanuddin University, Makassar, 90245, 23, Indonesia; j Faculty of Biology, Universitas Jenderal Soedirman, Purwokerto Utara, Banyumas, 53122, Central Java, Indonesia; k Iriomote Station, Tropical Biosphere Research Center, University of the Ryukyus, Taketomi, Okinawa, 907-1541, Japan

**Keywords:** Algae, Seaweed, Mangrove, Marine resource, Coral reef

## Abstract

Seaweeds are a valuable component of marine biodiversity that play multiple essential roles in Indonesia's coastal ecology and economy. This systematic review (1993–2023) aimed to provide an updated overview of seaweed distribution, biodiversity, cultivation, and industry in Indonesia. The literature search derived from major databases, Scopus, Web of Science (WoS) and ResearchGate (RG), and Google Scholar (GS) retrieved 794 studies, after removing 80 duplicates, identified 646 studies passed title and abstract screening that satisfied all criteria: Indonesia, seaweed, seaweed biodiversity and composition, which consisted of 80 exclusion studies. Full text screening decided 194 studies were selected based on the specific inclusion criteria (at least two criteria passed: seaweed distribution site, species, cultivation, and habitat). After additional filtering, 137 studies were included for extraction and analysis. We found that Indonesia is rich in seaweed biodiversity, with at least 325 identified species consisting of 103 Chlorophyceae (green algae), 167 Rhodophyceae (red algae), and 55 Phaeophyceae (brown algae), respectively. Seaweed distribution and abundance in Indonesia are influenced by environmental factors, including nutrients, grazing, competition, physical tolerance, light intensity, and degree of water circulation. Seaweed species are predominantly found in mangrove forests and coral reefs on the islands of Sumatra, Java, Kalimantan, and Sulawesi. This review provides an up-to-date and comprehensive overview of the distribution and biodiversity of seaweeds in Indonesia, highlighting the ecological, economic, and cultivation of marine resources. In addition, we identify knowledge gaps and areas for further research, which can inform sustainable seaweed management and utilization in Indonesia. This review also emphasizes the significance of this marine resource to Indonesia's environment and economy.

## Highlights

• This systematic review provided an updated of seaweed distribution, biodiversity, cultivation, and industry in Indonesia.
• Indonesia is rich in seaweed biodiversity, with 325 identified species.
• Seaweed species in Indonesia are a valuable natural resource of economic, cultural, and ecological significance.
• Long-term interests and sustainable environmental factors are considered when developing the economic potential of seaweed.

## 1 Introduction

Seaweed comprises a diverse group of photosynthetic macroalgae. Belonging to the Eukarya domain, seaweed is classified into the kingdoms Chromista (brown algae) and Plantae (green and red algae) based on the pigment molecules in the chloroplasts of each species [1,2]. The structural morphology of thallus macroalgae can be influenced by strong phenotypic plasticity, leading to variations that are dependent on environmental factors, such as temperature, salinity, nutrient availability, benthic interaction, and water motion [3].

Hydrocolloids are complex, non-digestible polysaccharide compounds, which are frequently used in various goods for direct or indirect human consumption [4,5]. Seaweed is an important source of hydrocolloids; consequently, the market value of traded seaweed has risen over the past decade from US $10 billion in 2013 [6,7] to US$ 15 billion in 2021 [8]. Approximately 83 % of seaweeds, known as ‘sea vegetables,’ are produced for human use [9]. The remainder is used in fertilizers, animal feed additives, medicinal ingredients [10], and biotechnological applications [11]. To cope with increased demand, global macroalgal production has risen by 5.7 % annually, reaching 18 million tons of macroalgae sourced from wild harvest and aquaculture in 2011 [7,12]. Global seaweed production has continued to increase exponentially, reaching 31.8 million tons in 2018, 34.6 million tons in 2019, and 50 million tons in 2020 [6].

In Indonesia, seaweed has become a major source of income for thousands of small-scale seaweed farmers [13], as it requires relatively less intensive capital and production inputs than other coastal aquaculture activities. Unsurprisingly, seaweed farming is frequently viewed as a valuable source of supplementary revenue for coastal communities [14]. *Eucheuma, Kappaphycus,* and *Gracilaria* are three commercially cultivated seaweeds found predominantly in Indonesia. National production of Indonesian seaweed has increased dramatically owing to the growing commercial demand for two hydrocolloids: agar and carrageenan [15]. The rapid increase in seaweed production and the number of farms to cope with the rising demand for seaweed products may encourage innovative breakthroughs in farming technology and new products. However, new farming technologies and goods can only be developed when investments are combined with significant efforts in scientific research [16].

The diversity of Indonesian seaweed has been well studied. However, details regarding Indonesian seaweed biodiversity, biology, ecology, and biochemical composition (nutritional value) are rarely available and sometimes incomplete. Consequently, the diversification of seaweed-farmed species and products is limited. Relevant studies are required to circumvent these limitations, which will 1) provide information on the type of species with potential for aquaculture, 2) identify the most suitable and favorable ecological conditions for optimal seaweed growth and 3) increase our understanding of seaweed to further guide and advance seaweed research and development in Indonesia. This systematic review aimed to provide an overview of the distribution and biodiversity of seaweed species in Indonesia, highlighting the ecological, economic, and cultural importance of this marine resource. In addition, the review identified knowledge gaps and areas for future research, which can inform the development of sustainable management, utilization, and industry of seaweed resources in Indonesia.

## 2 Methods

### 2.1 Review objective

The overarching review question is formulated as follows:

● What literature and data are available on seaweed biodiversity and future research in Indonesia?

The review questions for the secondary level are:

● Which seaweed species are the most commonly studied in Indonesia? Is there a trend observed in these studies?
● What approaches and indicators are used for seaweed culture and studies in Indonesia?
● What are the challenges and gaps in the monitoring of seaweed in Indonesia?
● Where are seaweed cultivations conducted in Indonesia?

### 2.2 Review scope

This systematic review focuses on studies that have reported on the biodiversity, habitat, and composition of seaweeds in Indonesia. Data were gathered from published literature obtained through searches of preselected bibliographic databases, including Scopus, Web of Science, Research Gate, and Google Scholar.

The Population, Intervention, Comparator, and Outcome (PICO) of the review was initially defined using an internationally accepted standard approach to systematic reviews [17,18]. Detailed descriptions of the PICO approach used in this systematic review are as follows:

● *Population*: Seaweed in Indonesia.
● *Intervention*: Any approaches and efforts used for seaweed habitat and species composition/diversity
● *Comparator*: Any indicators used to monitor biodiversity (transect and satellite)
● *Outcome*: Updated distribution, biodiversity, and future direction of seaweed

Most of the methodological steps in this systematic review follow previous systematic reviews in environmental science [19] and provide guidance for systematic reviewers within this scientific discipline [19].

### 2.3 Literature search

**Fig. 1:**
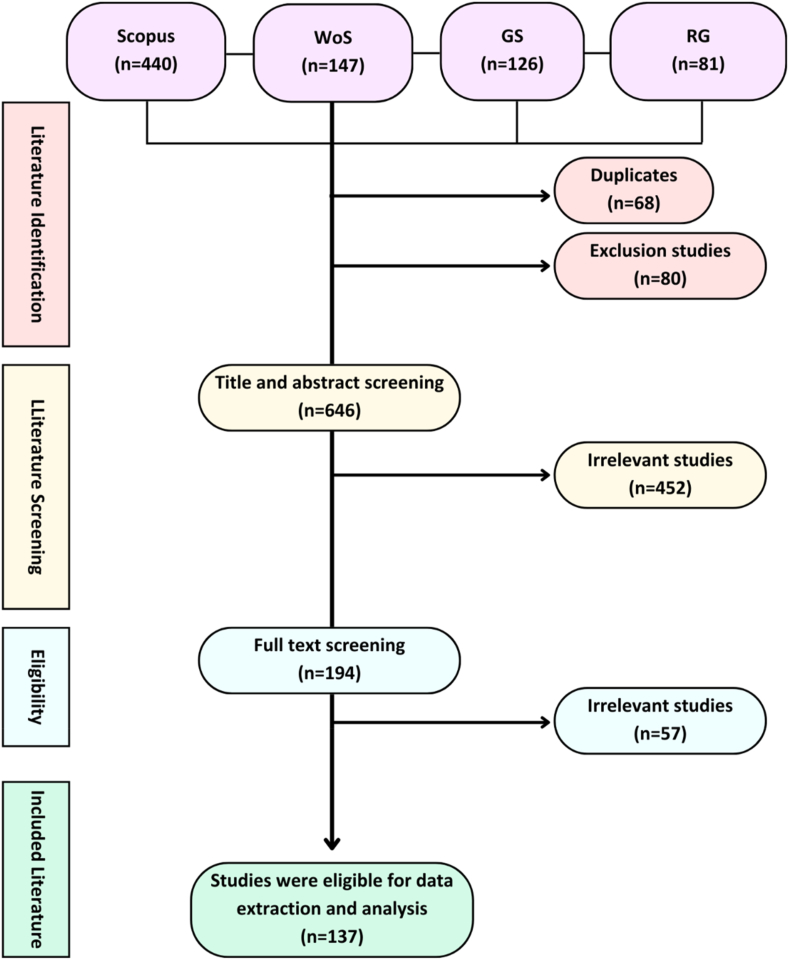
PRISMA flow diagram displaying the determination process of seaweed study subjects in Indonesia.

We created search strings by selecting pertinent terms in accordance with the PICO definitions before conducting the literature search in the bibliography databases. To prevent a limited search string and dearth of papers, we used terms from the Population and Intervention categories only. Literature searches in Scopus, Web of Science (WoS) and ResearchGate (RG) were conducted using English search strings. We used both English and Bahasa Indonesia search strings for Google Scholar (see the PRISMA flow chart in Table 1, Table 2 and Fig. 1 for thorough keyword and literary searches). We selected the first 65 papers and 61 papers of English GS and Bahasa Indonesia GS, in the most pertinent order to prevent the inclusion of irrelevant studies in the Google Scholar search results.

**Table 1:** Search string composition adopted from defined PICO with desired focus on studies from Indonesia in both English and Bahasa Indonesia.

Language	Geographical location	Population search terms	Intervention search terms
English	Indonesia	Seaweed OR macroalgae	seaweed habitat and species composition/diversity
Bahasa Indonesian	Indonesia	Rumput laut OR makroalga	Habitat rumput laut dan komposisi jenis atau diversitas

**Table 2:** Literature search record.

No	Database	Search string	Date of literature search	Search results
1	Scopus	((Indonesia AND seaweed) OR (habitat OR composition) AND (field AND satellite) OR (distribution AND biodiversity))	17/03/2024	440
2	Web of Science	((Indonesia AND seaweed) OR (habitat OR composition) AND (field AND satellite) OR (distribution AND biodiversity))	17/03/2024	147
3	Google Scholar English	((Indonesia AND seaweed) OR (habitat OR composition) AND (field AND satellite) OR (distribution AND biodiversity))	17/03/2024	65
4	Google Scholar Bahasa Indonesia	((Indonesia AND rumput laut) OR (habitat OR komposisi) AND (lapangan AND satelite) OR (distribusi AND biodiversitas))	17/13/2024	61
5	Research Gate	((Indonesia AND seaweed) OR (habitat OR composition) AND (field AND satellite) OR (distribution AND biodiversity))	17/03/2024	81

### 2.4 Literature screening

The inclusion criteria listed in Table 3 were used to determine the relevance of the published studies. Studies must satisfy the requirements of population, intervention, comparator, and results of interest for inclusion in the review. All studies underwent a two-stage screening procedure using a combination of title and abstract screening. To choose which articles met the scope of the review, the questions developed using the PICO model were employed at all screening stages.

**Table 3:** List of inclusion and exclusion criteria for papers using combined title-abstract and full-text screenings.

Screening stages	Questions	Screening outcome
Title and abstract screening	● Is the study located in Indonesia? ● Does the study focus on seaweed? ● Does the study present an assessment of seaweed biodiversity? ● Does the study present an assessment of seaweed composition?	Studies are included if all questions are satisfied
Full-text screening	● Does the study present the distribution of seaweed study sites? ● Does the study present any dataset related to seaweed species? ● Does the study consider the cultivation of seaweed? ● Does the study consider seaweed habitat? For included studies, additional follow-up open questions are asked to identify general information about the studies: ● Publication type (e.g., J: journal article, P: proceeding conference, T: thesis, B: book chapter, R: report) ● What is the type of the study? (L: Laboratory, R: review, RS: remote sensing, O: Opinion) ● What is the dataset presented in the study relevant to the review	Studies are included if at least two screening questions were met

### 2.5 Reporting and presentation

This systematic review's results are reported and presented following a standardized reporting methodology for systematic reviews of environmental studies [17,18] In addition to the report, a database comprising the results of the systematic review screening is provided.

## 3 Results

### 3.1 Publication screening

Fig. 1 shows the PRISMA flow diagram of the publication screening. In the initial phase, 794 papers were retrieved using the systematic literature search phrases listed in Table 1. Subsequently, 137 final papers were selected for further data extraction after literature screening, which included title, abstract, and full-text screening (based on the research topic relevance of the original papers). Overall, 17 % of the initially recognized publications in Scopus, WoS, Google Scholar, and ResearchGate publication databases were included in the extraction and analysis process. Major scholarly databases (Scopus and Google Scholar) and the results of the literature searches indicated that the publications were registered in duplicate (Fig. 1). The cutoff date for the literature search was March 17, 2024.

**Fig. 2:**
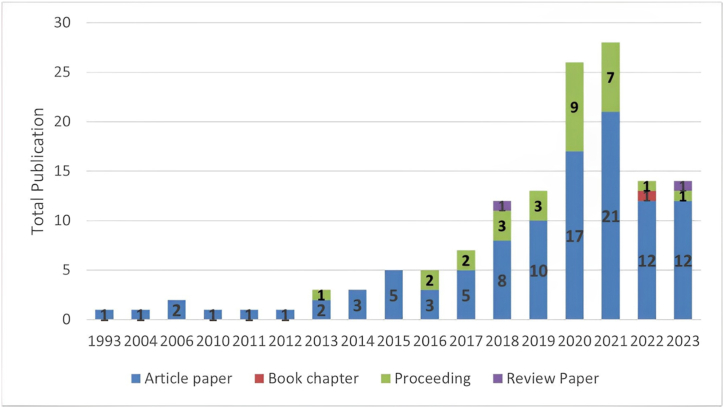
Publication trend of studies on seaweed biodiversity and distribution in Indonesia (year vs number and type of publication).

The number of publications on seaweed diversity and distribution in Indonesia will increase significantly from one in 1993 to 28 in 2021 (Fig. 2). The number of publications was low (did not exceed two) between 1993 and 2013. Subsequently, the number of published papers increased significantly from three in 2014 to 28 in 2021 and decreased to 14 in 2022 and 2023 (Fig. 2). Recently, the types of publications have become more diverse (journal articles, book chapters, proceedings, and review papers) than those published decades ago. Journal articles were the most dominant, followed by conference articles and review papers.

### 3.2 Seaweed distribution in Indonesia

This review revealed that Indonesia is rich in seaweed biodiversity, consisting of 325 species classified into 103 green algae (Supplementary Table 1), 167 red algae (Supplementary Table 2), and 55 brown algae (Supplementary Table 3). Tropical seaweed species are found in habitats such as coral reefs and mangrove forests. In addition, this review identified areas with high biodiversity, including the Seribu Islands, Spermonde Archipelago, Bali-Lombok Strait, Lesser Sunda Islands, Banda Sea, and Maluku Islands.

**Fig. 3:**
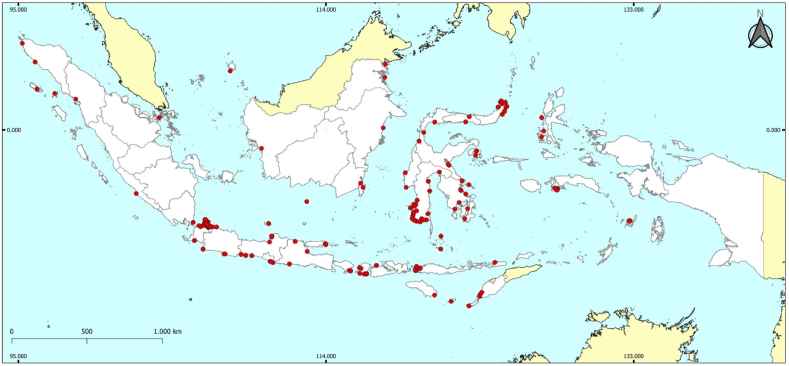
Distribution of seaweed study sites in Indonesia.

We identified 166 seaweed distribution sites in 24 Indonesian provinces: Aceh, Bali, Banten, Bengkulu, Central Java, Central Sulawesi, East Java, East Kalimantan, East Nusa Tenggara, Gorontalo, Jakarta, Lampung, Maluku, North Kalimantan, North Maluku, North Sumatra, the Riau Archipelago, South Kalimantan, South Sulawesi, Southeast Sulawesi, West Java, West Nusa Tenggara, West Sulawesi, and Yogyakarta (Fig. 3; Supplementary Tables 1–3). Jakarta had the most sites (30), followed by South Sulawesi (25), East Nusa Tenggara (17), North Sulawesi (10), and Central Sulawesi (nine). Notably, 30 sites in Jakarta belong to the Seribu Islands. Three provinces had only one site each: Lampung (South Lampung), North Kalimantan (Nunukan Island, Nunukan Regency), and North Sumatra (West Sorkam, Central Tapanuli) (Fig. 3). Seaweed in Bali was concentrated on Nusa Penida Island and Nusa Lembongan (Fig. 3). In East Nusa Tenggara, seaweed was found in Komodo National Park.

Most seaweed study sites are located on Java, Kalimantan, Sulawesi, and Lesser Sunda Island, with very limited data on seaweed distribution in Papua, Maluku, and Sumatra. This indicates that these areas, particularly Papua, are not well-documented. Considering the geographical barrier for biodiversity distribution represented by the Wallacea line, the Pacific zone/eastern part of Indonesia (Papua and Maluku) has unique biodiversity that is different from the Hindian zone/western region of Indonesia (Java, Sumatra, and Borneo) and transition zone (Sulawesi). The seaweed diversity presented in Fig. 3 is based on published literature obtained from the search strings and criteria in this study. The higher diversity of seaweed in certain areas compared to that of others in this study is simply because we did not find any published literature in that area that was relevant to or matched the search strings and criteria. However, more data and information on seaweed distribution are required to verify or improve our knowledge of seaweed distribution in these regions.

### 3.3 Seaweed species diversity

**Fig. 4:**
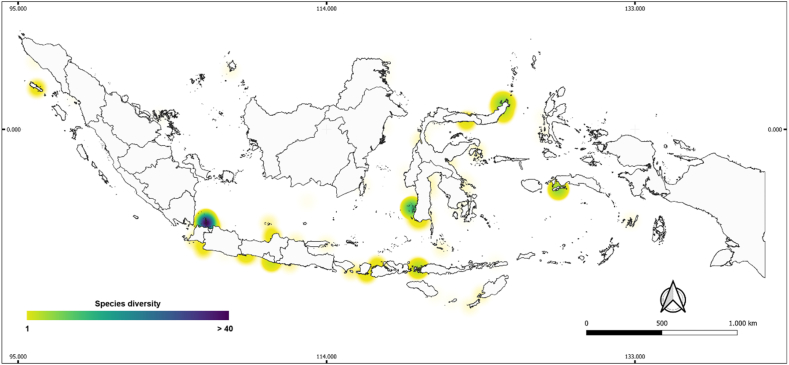
Seaweed species diversity from data obtained from systematic review papers in Indonesia.

We identified 10 sites within four provinces that have a high number of seaweed species in Indonesia (including one site with more than 40 species), namely, Seribu Islands in Jakarta province consisting of Air Island, Kotok Besar Island, Semak Daun Island, Tidung Besar Islands, Tidung Kecil Island, and Kotok Kecil Island; Jakarta (261 species); Spermonde Archipelago, South Sulawesi province (146 species); Butong and Hutumuri, Ambon, Maluku Province (80 species); and Mantehage Island, North Sulawesi province with 44 species (Fig. 4).

### 3.4 Seaweed cultivation

In 2019, the volume of seaweeds produced in Indonesia was approximately 9.66 million tons. After China, Indonesia is the second-largest seaweed producer in the world, contributing 38 % of the global seaweed market [20]. Besides supplying the market with seaweed-based products, the relatively recent potential benefit of seaweed to counter climate change has captured the attention of various research institutes studying the role of seaweed as a human-induced carbon sink [20]. In 2020, a total of 36 million tons (wet weight) of algae were produced, with 97 percent of it coming from aquaculture. The production of algae has undergone significant expansion in recent decades, increasing from 12 million tonnes in 2000 to 21 million tonnes in 2010. The growth in 2020 was a mere 2 percent when compared to 2019. Asian countries have solidified their position as significant producers, accounting for 97 percent of the overall algal production. In 2020, China and Indonesia were the top producers, contributing to the overall amount [12].

Indonesia is a significant producer and exporter of both raw and processed seaweed products in the international seaweed market. According to the Ministry of Marine Affairs and Fisheries (MMAF), Indonesia was one of the top producers of seaweed worldwide in 2021 (estimated 9.05 million tons of seaweed (live weight)) [20].

Seaweed production in Indonesia is spread across 23 provinces. The top five seaweed-producing provinces were South Sulawesi, East Nusa Tenggara, North Kalimantan, Central Sulawesi, and West Nusa Tenggara (Fig. 4).

**Fig. 5:**
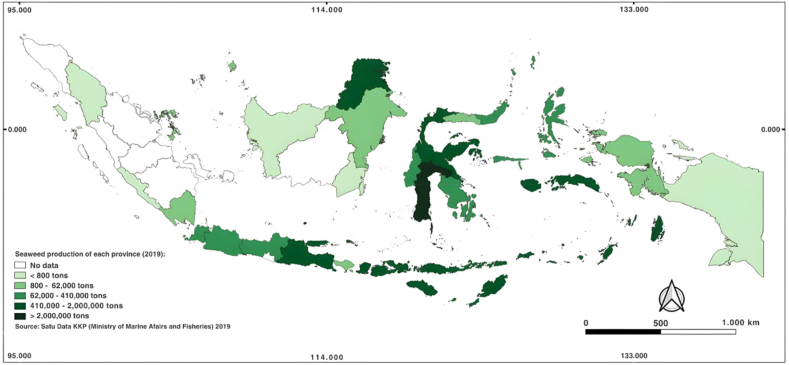
National seaweed farming production in 2019 [20].

Seaweed production in South Sulawesi reached more than 2.0 million tons (wet weight) in 2019 and slightly decreased to 1.63 million tons (wet weight) in 2020. Ranking second was East Nusa Tenggara, with a seaweed production of 1.03 million wet tons. North Kalimantan produced 441.1 thousand tons (wet weight) of seaweed, followed by Central Sulawesi (419.9 thousand tons, and West Nusa Tenggara (402.6 thousand tons (Fig. 5).

### 3.5 Seaweed industries

Seaweed-based hydrocolloid products contribute significantly to Indonesia's state income. Table 4 presents the export value (in both kilograms of dry material and USD) contributed by Indonesian seaweed and seaweed-based hydrocolloid products from 2020. Seaweed-based hydrocolloid products contribute 32 % of the total exports from SRC (Semi-refined carrageenan), Refined Carrageenan (RC), and agar. The total value in USD obtained from seaweed-based hydrocolloids reached 88 million in 2020, whereas dry material contributed 183 million USD.

**Table 4:** Export of Indonesian seaweed and seaweed-based hydrocolloids in 2020 [21].

Export in 2020 based on HS Code	Dry weight in Kg	Value in USD
*Eucheuma spinosum*	21,283,318	10,928,811
*Eucheuma cottonii*	97,187,191	131,294,886
*Sargassum*	1,365,418	322,826
Other seaweed	22,348,436	18,846,345
*Gelidium*	90,121	192,439
*Gracilaria*	39,250,253	21,802,593
SRC	11,411,681	71,643,273
RC	323,027	5,098,245
ATCC	464,504	2,786,349
Agar-agar	814,612	11,605,414

## 4 Discussion

In Indonesia, seaweed distribution is a complex and multifaceted topic that encompasses geographical, environmental, economic, and regulatory factors. Seaweed or ‘macroalgae’ are stenothermic marine benthic organisms found in tropical regions [35]. In general, the distribution and abundance of tropical macroalgae are affected by several biotic factors (e.g*.,* competition and grazing) [31] and abiotic factors (e.g., physical tolerance, light availability, and nutrients) [30].

Indonesia is an archipelagic country comprised of 17,000 islands with a 104,000 km tropical coastline [44]. The complex geographical characteristics of Indonesia's tropical coastline provide an optimal habitat for seaweeds [44]. For example, *Kappaphycus* seaweeds require surface sea temperatures of 22–33 °C [45]. Indonesia has with temperatures ranging from 21 to 33 °C, thus providing ideal conditions for *Kappaphycus* growth. Consequently, a variety of seaweed species occur along the coastlines of most Indonesian islands, with farming centers primarily concentrated in areas such as Sulawesi, Nusa Tenggara, Maluku, and Papua [13]. In addition, other coastal ecosystems, such as mangroves and seagrasses are strongly associated with seaweed occurrence. Macroalgae commonly occur in reef patches, such as reef flats and back reefs [27].

The base substrate types used by macroalgae as habitats can be distinguished into soft and hard substrates. Soft substrates, such as mud, sand, or a sand and silt mixture [28] are generally suitable for *Caulerpa*, *Gracillaria*, *Halimeda*, and *Hypnea* populations. Hard substrates, such as dead coral, rubble coral, and rocky outcrops, have been identified as preferred habitats for community structures of *Sargassum, Turbinaria*, and *Eucheuma* [29].

Coral-algal interactions depend largely on the physical, biological, and chemical properties of macroalgae [32]. Nutrient concentrations (nitrate, phosphate, and ammonia) in seawater are the most important water quality parameters related to macroalgae distribution and diversity in tropical waters [33], whereas microalgae biodiversity and abundance are strongly affected and tolerated by aquatic environmental factors such as temperature, salinity, and pH. Tropical macroalgae growth generally peaks at temperatures between 25 °C and 30 °C [34].

The practice of cultivating seaweed has deep cultural, economic, and environmental significance in Indonesia [13]. Community-based farming accounts for the vast majority of seaweed production in the country [38], thus providing livelihoods for many coastal communities. Many products can be produced from sea resources, such as seafood, raw materials for the chemical and pharmaceutical industries, cosmetics, fertilizers, and renewable energy (e.g., sea wave and wind energy). Therefore, seaweeds have good economic prospects— both locally and globally [36,37]. For example, cultivated seaweeds can be used to supply the base raw materials for various food products, such as sushi and nori, and cosmetics, organic fertilizers, and healthcare products. Seaweed farming is considered an environmentally friendly economic activity that increases seafood production and reduces pressure on natural fish stocks [37]. In addition, seaweed has the potential to be developed as a source of renewable energy such as ocean wave energy and sea breezes. Ocean wave energy can be generated from differences in height and low seawater, whereas sea wind energy can be generated from air currents that occur above the sea level [39,40]. Continued growth and success of the industry will depend on sustainable practices, technological advancements, and ongoing efforts to address challenges, such as disease management and environmental conservation.

Most studies concerning seaweed in Indonesia appear to focus on its use, such as food additives [15], cosmetic purposes [22], nutrient content [23,24], biostimulants, and animal feed [25]. The global demand for seaweed-based products such as carrageenan, alginate, and agar is increasing in various industries, including food, pharmaceuticals, cosmetics, and biofuels. Indonesia is the largest producer of *Kappaphycus*, from which three hydrocolloid products are courced: kappa carrageenan, iota carrageenan, and agar, which are extracted from *Kappaphycus alvarezzi* (known as *Eucheuma cottonii*), *E. denticulatum*, and *E. spinosum* and *Gracilaria* sp., respectively. All three species are classified as red algae, Rhodophyta [43]. The major routes of marketing hydrocolloid products consist of (1) direct delivery of dry seaweed material to a finished hydrocolloid, and subsequently to the end user; and (2) blenders: first, from dry seaweed material to a finished hydrocolloid; second, to a blending house; and third, onto the end user as a proprietary blend. The first route is the simplest model in which the hydrocolloid is normally sold as a specified product [15].

As one of the world's largest producers of seaweed, Indonesia strongly assists in meeting the growing global demand, enhancing economic growth and expanding export opportunities [26]. Indonesia has great prospect to further increased its seaweed production capacity. With approximately 12.3 million hectare of seaweed culture potential area, only about 102 ha or 0.8 % is used for seaweed cultivation. In order to deal with this situation, recently the Indonesian government through the Ministry of Marine Affairs and Fisheries (MMAF) and Coordinating Ministry for Maritime and Investment Affairs (CMMIA) implemented the “Seaweed Downstreaming Policy” to increase the capacity and improve the management and commercial aspect of the seaweed industry in Indonesia. This policy includes several aspects such as initiating the seaweed cultivation pilot project in five different location (Buleleng, Wakatobi, Maluku Tenggara, Rote Ndao, and Nusa Tenggara Barat) and development of seaweed-based biofuel and crude oil as well as biogradeable plastic materials [41,42].

However, there are challenges in developing the economic potential of seaweed. One is the problem of sustainable management of marine resources and reduction in the negative environmental impacts of seaweed farming. For example, vast seaweed farming rafts in one location have created conflicts between coastal and marine traffic. The use of plastics and microplastic contamination of farmed seaweed is an emerging challenge in developing sustainable seaweed aquaculture in Indonesia. Therefore, future studies on the economic potential of seaweed must comprehensively and studiously consider its sociocultural and environmental impacts.

## 5 Future direction

Seaweed species in Indonesia are a valuable natural resource of economic, cultural, and ecological significance, which provide opportunities for sustainable economic development, biodiversity conservation, and scientific research. However, the industry must address potential future challenges and implement sustainable practices to conserve seaweed species remain healthy and their diversity can be sustainably managed. Indonesia is globally in a competitive position in the hydrocolloid market, as the global carrageenan industry is remarkable with high prospects for future growth.

To maintain its share in the global market, Indonesia must overcome existing challenges and threats owing to market dynamics and domestic issues. One of the threats to the market is the presence of non-seaweed hydrocolloid products originating from terrestrial plants, chemicals, and bacteria. Another highlighted threat is the climate crisis, which leads to uncertainty in the planting season for seaweed farming. Regarding domestic issues, Indonesia still has a critical problem with the supply chain system, causing volatile prices for dry material and overlapping policies or regulations. These domestic issues may lead to noncompetitive products and supply instability. To overcome these challenges and threats, every seaweed stakeholder needs to collaborate with governments, the private sector, academics, researchers, and even NGOs. Thus, the sustainability of the Indonesian seaweed industry can be achieved.

## 6 Conclusion

The distribution and biodiversity of seaweeds in Indonesia are thoroughly covered in this systematic review. This review also emphasizes the significance of this marine resource to Indonesia's environment and economy. This analysis recommends areas for additional research and identifies knowledge gaps that could help Indonesia create sustainable methods for managing and utilizing seaweed resources. Therefore, it is important to consider both long-term interests and sustainable environmental factors when developing the economic potential of seaweed.

## Fundings

This work was supported under Basic Research Collaboration Scheme 2022–2024 (e-Asia Joint Research Program) (079/UN5.2.3.1/PPM/KP-DRTPM/L/2022) from the 10.13039/100009950Ministry of Education, Culture, Research and Technology of Government of Indonesia and under 10.13039/501100013375Universitas Sumatera Utara for Talenta Grant No. 63/UN5.2.3.1/PPM/SPP-TALENTA USU/2020, and under EQUITY Scheme from 10.13039/501100013375Universitas Sumatera Utara to Center of Excellence for Mangrove 2023–2024 (91/UN5.2.3.1/PPM/KPEP/2023) (MB).

## Ethical clearance statement

Not applicable.

## Data availability statement

The authors don't have any research data outside the submitted manuscript file.

## CRediT authorship contribution statement

**Mohammad Basyuni:** Writing – review & editing, Writing – original draft, Visualization, Validation, Supervision, Project administration, Methodology, Funding acquisition, Formal analysis, Data curation, Conceptualization. **Maya Puspita:** Writing – review & editing, Methodology, Investigation, Data curation. **Rinny Rahmania:** Writing – review & editing, Visualization, Validation, Supervision. **Hatim Albasri:** Writing – review & editing, Validation. **Indra Pratama:** Writing – review & editing. **Dini Purbani:** Writing – review & editing. **A.A. Aznawi:** Writing – review & editing, Visualization, Investigation. **Alfian Mubaraq:** Writing – review & editing, Validation, Formal analysis, Data curation. **Shofiyah S. Al Mustaniroh:** Writing – review & editing, Resources. **Firman Menne:** Writing – review & editing. **Yulizar Ihrami Rahmila:** Writing – review & editing. **Severino G. Salmo:** Writing – review & editing. **Arida Susilowati:** Writing – review & editing. **Siti H. Larekeng:** Writing – review & editing, Supervision, Formal analysis. **Erwin Ardli:** Writing – review & editing. **Tadashi Kajita:** Writing – review & editing, Funding acquisition.

## Declaration of competing interest

The authors declare that they have no known competing financial interests or personal relationships that could have appeared to influence the work reported in this paper.
