# Supplementary material for: Current biodiversity status, distribution, and prospects of seaweed in Indonesia: A systematic review
Source: Heliyon. 2024 May 10;10(10):e31073. doi: 10.1016/j.heliyon.2024.e31073 (PMC11109829; doi:10.1016/j.heliyon.2024.e31073)
Supplement: Multimedia component 1 [file mmc1.docx]

Supplementary information for

**A systematic review of the current biodiversity status, distribution, and further prospects of seaweed in Indonesia**

Mohammad Basyuni^a,b*^, Maya Puspita^c^, Rinny Rahmania^d^, Hatim Albasri^e^, Indra Pratama^e^, Dini Purbani^f^, A. A. Aznawi^a^, Alfian Mubaraq^a^, Shofiyah S. Al Mustaniroh^a^, Firman Menne^g^, Yulizar Ihrami Rahmila^d^, Severino G. Salmo III^h^, Arida Susilowati^a,b^, Siti H. Larekekng^i^, Erwin Ardlj^j^, Tadashi Kajita^k^

^a^Center of Excellence for Mangrove, Universitas Sumatera Utara, Medan 20155, Indonesia

^b^Department of Forestry, Faculty of Forestry, Universitas Sumatera Utara, Medan 20155, Indonesia

^c^Asosiasi Rumput Laut Indonesia, Jakarta, Indonesia,

^d^Research Center for Ecology and Ethnobiology, National Research and Innovation Agency, Cibinong 16911, Indonesia

^e^Research Center for Fisheries, National Research and Innovation Agency, Cibinong 16911, Indonesia

^f^ Research Center for Conservation of Marine Resources and Inland Waters, Cibinong 16911, Indonesia

^g^Department of Accounting, Faculty of Economics and Business, Universitas Bosowa, Makassar, Indonesia.

^h^ Institute of Biology, College of Science, University of the Philippines Diliman, Quezon City, Philippines

I Biodiversity Research Group, Faculty of Forestry, Hasanuddin University, Makassar 90245, Indonesia

^j^ Faculty of Biology, Universitas Jenderal Soedirman, Purwokerto Utara, Banyumas 53122, Central Java, Indonesia

^k^Iriomote Station, Tropical Biosphere Research Center, University of the Ryukyus, Taketomi, Okinawa, 907-1541, Japan

*Corresponding author

Mohammad Basyuni, E-mail: m.basyuni@usu.ac.id

Supplementary Table 1. Macroalgae from Chlorophyceae (green algae)

Supplementary Table 2. Macroalgae from Rhodophyceae (red algae)

Supplementary Table 3. Macroalgae from Phaeophyceae (brown algae)

Supplementary Table 1. Macroalgae from Chlorophyceae (green algae)

| Chlorophyceae | Site | References |  |
| --- | --- | --- | --- |
| *Avrainvillea longicaulis* | Spermonde Archipelago, South Sulawesi | [146] |  |
| *Avrainvillea sp.* | Kotok Kecil and Putri Island, Seribu Island, Jakarta | [66] |  |
| *A. amadelpha* | Simeulue Island, Aceh  Spermonde Archipelago, South Sulawesi  West coast Banda Aceh, Aceh | [68]  [146]  [161] |  |
| *A. erecta* | Minahasa (Kampung Ambong, Poopoh, and Tumbak, North Sulawesi  Kotok Kecil and Putri Island, Seribu Island, Jakarta  Mantehage Island, North Sulawesi | [89]  [66]  [87] |  |
| *A. nigricans* | Spermonde Archipelago, South Sulawesi | [146] |  |
| 1. *gardineri* | Spermonde Archipelago, South Sulawesi | [146] |  |
| *A. lacerata* | Kotok Kecil and Putri Island, Seribu Islands, Jakarta  Spermonde Archipelago, South Sulawes | [66]  [146] |  |
| *A. obscura* | Spermonde Archipelago, South Sulawesi | [146] |  |
| *Acetabularia dentata* | Poopoh and Kampung Ambong, Minahasa, North Sulawesi  Spermonde Archipelago, South Sulawesi | [89]  [146] |  |
| *Bornetella nitida* | Seribu Island (Island of Payung Kecil, Tidung Kecil, Tidung Besar, Air, Semak Daun, Kotok Besar, Panjang, Belanda) Jakarta  Pari Island, Seribu Island, Jakarta  Spermonde Archipelago, South Sulawesi  Ujung Genteng, Sukabumi, West Java  Labuhanbua, West Nusa Tenggara | [66]  [138]  [146]  [70]; [69] |  |
| *Bornetella oligosphora* | Kampung Ambong, Poopoh and Tumbak, Minahasa Regency, North Sulawesi | [89]; [88] |  |
| *Bornetella sphaerica* | Kampung Ambong, Poopoh and Tumbak, Minahasa Regency, North Sulawesi  Mantehage Island, North Sulawesi | [89]; [88]  [87] |  |
| *Boodlea composita* | Tanjung Merah, Bitung, North Sulawesi  Minahasa Regency (Kampung Ambong, Poopoh and Tumbak) North Sulawesi  Seribu Island (Island of Ayer Besar, Lancang Besar, Tidung Kecil, Tidung Besar, Air, Semak Daun, Kotok Kecil, Kotok Besar, Peniki, Kelapa, Panjang, Sepa Besar) Jakarta.  Spermonde Archipelago, South Sulawesi  Padar dan Muntia, Komodo National Park, East Nusa Tenggara  Taka Bonerate, Selayar Island, South Sulawesi | [89]; [88]  [66]  [146]  [157]  [143]  [171] |  |
| *Bryopsis pennata* | Seribu Island (Island of Bidadari, Onrust, Kelor, Ayer Besar, Damar Kecil, Untung Jawa, Payung Kecil, Tidung Kecil) Jakarta  Porok Beach, Gunungkidul, Yogyakarta | [66]  [62] |  |
| *B. thwaitesii* | West coast Banda Aceh, Aceh | [161] |  |
| *B. plumosa* | West coast Banda Aceh, Aceh | [161] |  |
| *Boergesenia forbesii* | Kampung Ambong, Poopoh and Tumbak, Minahasa Regency, North Sulawesi  Seribu Island (Island of Tikus Kecil, Tidung Kecil Air, Peniki, Belanda, Hantu Kecil, Hantu Besar, Pari) Jakarta  Karapyak Beach, Pangandaran, West Java  Spermonde Archipelago, South Sulawesi  Mangaitan, Komodo National Park, East Nusa Tenggara  Nusa Penida Island, Bali  Mantehage Island, North Sulawesi  Permisan Beach Nusakambangan Island, Cilacap, Central Java  West coast Banda Aceh, Aceh  Pari Island, Seribu Island, Jakarta  Taka Bonerate, Selayar Island, South Sulawesi  Sepang Bay, Bengkulu | [89]; [88]  [66]  [138]; [74]  [63]  [146]  [157]  [117]; [71]  [87]  [117]; [71]  [161]  [163]  [171]  [158] |  |
| *C. crassa* | Taka Bonerate, Selayar Island, South Sulawesi | [171] |  |
| *Chaetomorpha sp.* | Seribu Island (Island of Payung Kecil, Tidung Besar, Kotok Besar, Peniki) Jakarta  Sepanjang Beach, Gunung Kidul, Yogyakarta | [66]  [132] |  |
| *Chaetomorpha spiralis* | Mantehage Island, North Sulawesi | [87] |  |
| *Codium simulans* | Porok Beach and Greweng coast, Gunungkidul, Yogyakarta | [62] |  |
| *C. edule* | Onrust Island, Seribu Island, Jakarta  Spermonde Archipelago, South Sulawesi  Komodo National Park (Setuga, Padar, and Mangaitan) East Nusa Tenggara  Krakal Beach, Gunungkidul, Yogyakarta  Olibuu, Paguyaman, Boalemo, Gorontalo | [66]  [146]  [157]  [114]  [148] |  |
| *C. bartletti* | Spermonde Archipelago, South Sulawesi | [146] |  |
| *C. geppi* | Spermonde Archipelago, South Sulawesi | [146] |  |
| *C. harveyi* | Spermonde Archipelago, South Sulawesi | [146] |  |
| *C. decorticatum* | Papagarang, Komodo National Park, East Nusa Tenggara  Ambon (Suli, Hutumuri, Rutong, and Leahari) Maluku  Camara Beach, Lombok, west Nusa Tenggara  Olibuu, Paguyaman, Boalemo, Gorontalo | [157]  [109]  [136]  [148] |  |
| *C. arabicum* | Semak Daun Island, Seribu Island, Jakarta  Spermonde Archipelago, South Sulawesi  Setuga and Mangaitan, Komodo National Park, East Nusa Tenggara | [66]  [146]  [157] |  |
| *C. repens* | Sepanjang Beach, Gunungkidul, Yogyakarta | [132] |  |
| *C. tomentosum* | Menganti Beach, Kebumen, Central Java | [162] |  |
| *Chaetomorpha antenia* | Greweng coast, Yogyakarta  Permisan Beach Nusakambangan Island, Cilacap, Central Java  Simeulue Island, Aceh  West coast Banda Aceh, Aceh | [62]  [117]; [71]  [68]  [161] |  |
| *C. crassa* | Kampung Ambon, North Minahasa, Poopoh, Tumbak, Minahasa, North Sulawesi  Spermonde Archipelago, South Sulawesi  Padar, Komodo National Park, East Nusa Tenggara  Suli, Hutumuri, Hutong, Leahari Ambon, Maluku  Mantehage Island, North Sulawesi  Kora-kora, Minahasa, North Sulawesi  Arungkeke, Janeponto, South Sulawesi  Permisan Beach Nusakambangan Island, Cilacap, Central java  Ujung Genteng, Sukabumi, West Jawa  Labuhanbua, Sumbawa, West Nusa Tenggara  Krakal Beach, Yogyakarta  Olibuu, Boalemo, Gorontalo | [89]  [146]  [157]  [109]  [87]  [143]  [98]  [117]; [71]  [70]  [70]; [69]  [114]  [148] |  |
| *C. linum* | Porok Beach, Gunungkidul, Yogyakarta  West coast Banda Aceh, Aceh | [62]  [161] |  |
| *Cladophora catenata* | Kampung Ambong, North Sulawesi  Poopoh, Minahasa, North Sulawesi  Tumbak, Minahasa, North Sulawesi  Spermonde Archipelago, South Sulawesi  Padar, Komodo National Park  Suli, Hutumuri, Hutong, Leahari Ambon, Maluku  Mantehage Island, North Sulawesi  Kora-kora, Minahasa, North Sulawesi  Arungkeke, Janeponto, South Sulawesi  Nusakambangan, Central Java  Ujung Genteng, West Java  Labuhanbua, Sumbawa, West Nusa Tenggara  Krakal Beach, Yogyakarta  Olibuu, Boalemo, Gorontalo  Krakal Beach, Yogyakarta | [89]; [88]  [146]  [157]  [109]  [65]  [143]  [98]  [117]; [71]  [70]  [70]; [69]  [114]  [148]  [114] |  |
| *C. sericea* | West coast Banda Aceh, Aceh | [161] |  |
| *Cladophora sp.* | Krakal Beach, Gunung Kidul, Yogyakarta | [114] |  |
| *Caulerpa serrulata* | Kampung Ambong, North Sulawesi  Poopoh, Minahasa, North Sulawesi  Tumbak, Minahasa, North Sulawesi  Seribu Islands (Islands of Bokor, Tikus, Payung Kecil, Tidung Kecil, Tidung Besar, Air, Kotok Besar, Kotok Kecil, Peniki, Kelapa, Panjang, Belanda, Putri, Sepa Besar, and Hantu Besar)  Mangaitan, East Nusa Tenggara  Spermonde Archipelago, South Sulawesi  Mantehage Island, North Sulawesi  Pari Island, Seribu Island, Jakarta | [89]; [88]  [66]  [157]  [146]  [87]  [163] |  |
| *C. racemosa* | Kampung Ambong and Poopoh, North Sulawesi  Tumbak, Minahasa, North Sulawesi  Seribu Islands (Islands of Onrust, Kelor, Ayer Besar, Damar Besar and Kecil, Untung Jawa, Bokor, Dapur, Lancang Besar, Tikus, Payung Kecil, Tidung Kecil, Tidung Besar, Air, Semak Daun, Kotok Besar, Kotok Kecil, Peniki, Kelapa, Panjang, Belanda, Putri, Sepa Besar, Jukung, Hantu Kecil, Hantu Besar, and Pari) Jakarta  Spermonde Archipelago, South Sulawesi  Setuga, Mangaitan, Komodo National Park, East Nusa Tenggara  Mantehage Island, North Sulawesi  Nature, Riau Island  Prigi Bay, Trenggalek, East Java  Olibuu, Boalemo, Gorontalo  West coast Banda Aceh, Aceh  Pari Island, Seribu Island, Jakarta  Menganti Beach, Kebumen, Central Jawa  Taka Bonerate, Selayar Island, South Sulawesi  Palang Coast, Tuban, East Java | [89]  [88]  [66]  [24]  [126]  [146]  [157]  [65]  [82-83]  [84]  [148]  [161]  [163]  [162]  [171]  [168] |  |
| *C. subserrata* | Spermonde archiepalgo, South Sulawesi | [146] |  |
| *C. sertularioides* | Kampung Ambon, North Minahasa, Poopoh, Tumbak, Minahasa, North Sulawesi  Seribu Islands (Tikus, Tidung Kecil, Air, Semak Daun, Kotok Besar, Kotok Kecil, Peniki, Kelapa, Belanda, Sepa Besar, Hantu Kecil, Hantu Besar;  Mantehage, North Sulawesi  West coast Banda Aceh, Aceh  Pari Island, Seribu Island, Jakarta | [89]  [66]  [87]  [161]  [163] |  |
| *C. lentillifera* | Seribu Islands (Untung Jawa, Lancang Besar, Kotok Besar, Kotok Kecil, Panjang Island, Putri Island  Spermonde archiepalgo, South Sulawesi  Mantehage, North Sulawesi  Simeulue island  Letman village, Kei Island  West coast Banda Aceh, Aceh  Menganti Beach, Kebumen, Central Jawa  Palang Coast, Tuban, East Java | [66]  [146]  [87]  [68]  [144]  [161]  [162]  [168] |  |
| *C. opposita* | Spermonde Archipelago, South Sulawesi | [146] |  |
| *C. brachypus* | Seribu Islands (Air, Semak Daun, Panjang, Belanda, Sepa Besar)  Spermonde Archipelago, South Sulawesi | [66]  [146] |  |
| *C. buginense* | Spermonde Archipelago, South Sulawesi | [146] |  |
| *C. taxifolia* | Poopoh, Minahasa, North Sulawesi  Nusakambangan Island, Central Java  Menganti Beach, Kebumen, Central Jawa  Palang Coast, Tuban, East Java | [89]  [117]; [71]  [162]  [168] |  |
| *C. manorensis* | Spermonde Archipelago, South Sulawesi | [146] |  |
| *C. scalpelliformis* | Spermonde Archipelago, South Sulawesi | [146] |  |
| *C. lessonii* | Seribu Island (Island of Bokor, Tikus, Payung Kecil, Tidung Kecil, Tidung Besar, Air, Kotok Besar, Kotok Kecil, Peniki, Kelapa, Panjang, Belanda, Putri, Sepa Besar, Hantu Besar) Jakarta  Spermonde Archipelago, South Sulawesi | [66]  [146] |  |
| *C. elongata* | Belanda Island, Seribu Island, Jakarta  Spermonde Archipelago, South Sulawesi | [66]  [146] |  |
| *C. verticulata* | Spermonde Archipelago, South Sulawesi | [146] |  |
| *C. cupressoides* | Seribu Island (Island of Tikus, Tidung Kecil, Tidung Besar, Air, Kotok Besar, Kotok Kecil, Peniki, Kelapa, Panjang, Belanda, Sepa Besar) Jakarta  Pari Island, Seribu Island, Jakarta  Spermonde Archipelago, South Sulawesi  Taka Bonerate, Selayar Island, South Sulawesi | [66]  [138]  [146]  [171] |  |
| *C. cylindracea* | Palang Coast, Tuban, East Java | [168] |  |
| *C. peltata* | Palang Coast, Tuban, East Java | [168] |  |
| *Caulerpa Sp.* | Pari Island, Seribu Island, Jakarta | [163] |  |
| *Chlorodesmis fastigiata* | Seribu Islands (Island of Lancang Besar, Kotok Besar, Kotok Kecil, Jukung) Jakarta  Spermonde Archipelago, South Sulawesi | [66]  [146] |  |
| *Chlorodesmis spp.* | Seribu Island (Island of Lancang, Kotok Besar, Kotok Kecil, Jukung) Jakarta | [66] |  |
| *C. hildebrandtii* | Seribu Island (Island of Lancang Besar, Kotok Besar, Kotok Kecil, Jukung) Jakarta  Spermonde Archipelago, South Sulawesi  Ambon (Suli, Hutumuri, Rutong, and Leahari) Maluku | [66]  [146]  [109] |  |
| *Dictyosphaeria cavernosa* | Kampung Ambon, North Minahasa,  Poopoh, Tumbak, Minahasa;  Spermonde archiepalgo, South Sulawesi  Komodo National Park (Setuga, Karang Makassar, Padar) | [89]  [146]  [157] |  |
| *Dictyosphaeria spp.* | Seribu Island (Island of Damar Kecil, Bokor, Tikus, Tidung Kecil, Tidung Besar, Air, Semak Daun, Kotok Besar, Kotok Kecil, Peniki, Kelapa, Panjang, Belanda) Jakarta  Pari Island, Seribu Island, Jakarta | [66]  [163] |  |
| *D. versluysi* | Spermonde Archipelago, South Sulawesi | [146] |  |
| *Enteromorpha clathrata* | Spermonde Archipelago, South Sulawesi | [146] |  |
| *E. compressa* | Libukang Island, Jenoponto, South Sulawesi  Spermonde Archipelago, South Sulawesi | [130]  [146] |  |
| *E. intestinalis* | Spermonde Archipelago, South Sulawesi  Arungkeke waters, South Sulawesi  Permisan Beach Nusakambangan Island | [146]  [98]  [117]; [71] |  |
| *Halimeda copiosa* | Spermonde Archipelago, South Sulawesi | [146] |  |
| *Halimeda sp.* | Ujung Genteng, Sukabumi, West Java  Labuhanbua, Sumbawa, West Nusa Tenggara | [81]; [70]  [70]; [69] |  |
| *Halimeda spp.* | Seribu Island (Island of Damar Besar, Untung Jawa, Bokor, Dapur, Lancang Besar, Payung Kecil, Tidung Kecil, Tidung Besar, Semak Daun, Kotok Besar, Kotok Kecil, Peniki, Kelapa, Panjang, Belanda, Putri, Sepa Besar, Jukung, Hantu Besar, Hantu Kecil) Jakarta | [66] |  |
| *H. cylindracea* | Spermonde Archipelago, South Sulawesi  Mantehage Island, North Sulawesi  Taka Bonerate, Selayar Island, South Sulawesi | [146]  [87]  [171] |  |
| *H. simulans* | Spermonde Archipelago, South Sulawesi  Ujung genteng, Sukabumi, West Java  Labuhanbua, West Nusa Tenggara | [146]  [81]  [70]; [69] |  |
| *H. macroloba* | Tanjung Merah, Bitung, North Sumatra  Kampung Ambong, Poopoh, and Tumbak, Minahasa, North Sulawesi  Pari Island, Seribu Island, Jakarta  Spermonde Archipelago, South Sulawesi  Komodo National Park (Setuga, Padar Kecil, Papagarang, and Muntia) East Nusa Tenggara  Ambon (Suli, Hutumuri, Rutong, and Leahari) Maluku  Mentahage, North Sulawesi  Kora-kora, Minahasa, North Sulawesi  Bandengan Beach, Jepara, Central Java  Karimunjawa Island, Central Java  Nain Island, Minahasa, North Sulawesi  Awur Bay, Jepara, Central Java  Ujung Genteng, Sukabumi, West Java  Labuhanbua, Sumbawa, West Nusa Tenggara  Prigi Bay, Trenggalek, East Java  Olibuu, Paguyaman, Baelemo, Gorontalo  West coast Banda Aceh, Aceh  Pari Island, Seribu Island, Jakarta  Taka Bonerate, Selayar Island, South Sulawesi  Sepang Bay, Bengkulu | [74]; [143]  [89]; [88]  [127]; [24] [126]  [146]  [157]  [109]  [89]  [143]  [49]  [118]  [124]  [114]  [81]  [70]; [69]  [84]  [148]  [161]  [163]  [168]  [158] |  |
| *H. melanesica* | Spermonde Archipelago, South Sulawesi | [146] |  |
| *H. distorta* | Spermonde Archipelago, South Sulawesi  Olibuu, Paguyaman, Baelemo, Gorontalo | [146]  [148] |  |
| *H. opuntia* | Tanjung Merah, Bitung, North Sumatra  Kampung Ambong, Poopoh, and Tumbak, Minahasa, North Sulawesi  Pari Island, Seribu Island, Jakarta  Spermonde Archipelago, South Sulawesi  Komodo National Park (Setuga, Padar Kecil, Padar, Mangaitan, and Muntia) East Nusa Tenggara  Ambon (Suli, Hutumuri, Rutong, and Leahari) Maluku  Binuangeun, Banten  Mentahage, North Sulawesi  Kora-kora, Minahasa, North Sulawesi  Bandengan Beach, Jepara, Central Java  South Lampung, Lampung  East Lombok, West Nusa Tenggara  Awur Bay, Jepara, Central Java  South Konawe, Southeast Sulawesi  Labuhanbua, Sumbawa, West Nusa Tenggara  Prigi Bay, Trenggalek, East Java  Olibuu, Paguyaman, Baelemo, Gorontalo  Simeulue Island, Aceh  West coast Banda Aceh, Aceh  Pari Island, Seribu Island, Jakarta  Palang Coast, Tuban, East Java  Sepang Bay, Bengkulu | [74]; [143]  [89]; [88]  [138]  [146]  [157]  [109]  [104]  [87]  [143]  [49]  [104]  [104]  [114]  [104]  [70]; [69]  [84]  [148]  [68]  [161]  [163]  [168]  [158] |  |
| *H. gracilis* | Spermonde Archipelago, South Sulawesi  Pari Island, Seribu Island, Jakarta | [146]  [163] |  |
| *H. micronesica* | Spermonde Archipelago, South Sulawesi  Ujung Genteng, Sukabumi, West Java  Labuanbua, Sumbawa, West Nusa Tenggara  Olibuu, Paguyaman, Boalemo (Gorontalo)  Pari Island, Seribu Island, Jakarta  Taka Bonerate, Selayar Island, South Sulawesi | [146]  [70]; [81]  [70]; [69]  [148]  [163]  [171] |  |
| *H. gigas* | Spermonde Archipelago, South Sulawesi | [146] |  |
| *H. macrophysa* | Pari Island, Seribu Island, Jakarta  Spermonde Archipelago, South Sulawesi | [138]  [146] |  |
| *H. discoidea* | Spermonde Archipelago, South Sulawesi  Mentahage, North Sulawesi  Simeulue Island, Aceh  Olibuu, Paguyaman, Baelemo, Gorontalo | [146]  [87]  [68]  [148] |  |
| *H. taenicola* | Spermonde Archipelago, South Sulawesi | [146] |  |
| *H. tuna* | Spermonde Archipelago, South Sulawesi  Mentahage, North Sulawesi | [146]  [87] |  |
| *H. minima* | Spermonde Archipelago, South Sulawesi | [146] |  |
| *H. maxima* | Taka Bonerate, Selayar Island, South Sulawesi | [171] |  |
| *Neomeris annulata* | Kampung Ambong, Poopoh, and Tumbak, Minahasa, North Sulawesi  Ambon (Suli, Hutumuri, Rutong, and Leahari) Maluku  Spermonde Archipelago, South Sulawesi  Mantehage Island, North Sulawesi  Palang Coast, Tuban, East Java | [89]; [88]  [109]  [146]  [87]  [168] |  |
| *N. vanbosseae* | Spermonde Archipelago, South Sulawesi | [146] |  |
| *Rhipidosiphon javensis* | Seribu Islands (Island of Kelor, Ayer Besar, Untung Jawa, Bokor, Tikus) Jakarta  Spermonde Archipelago, South Sulawesi | [66]  [146] |  |
| *Rhipilia orientalis* | Spermonde Archipelago, South Sulawesi | [146] |  |
| *Rhipilia spp.* | Payung Kecil and Semak Daun Island, Seribu Island, Jakarta | [66] |  |
| *Tydemania expeditionis* | Seribu Island (Island of Payung Kecil, Tidung Kecil, Air, Kotok Besar, Kotok Kecil, Peniki, Panjang, Putri, Sepa Besar, Jukung, Hantu Kecil) Jakarta  Spermonde Archipelago, South Sulawesi | [66]  [146] |  |
| *Udotea orientalis* | Spermonde Archipelago, South Sulawesi | [146] |  |
| *U. glaucescens* | Spermonde Archipelago, South Sulawesi | [146] |  |
| *U. flabellum* | Spermonde Archipelago, South Sulawesi  Permisan Beach Nusakambangan Island, Cilacap, Central Java | [146]  [117]; [71] |  |
| *U. argentea* | Spermonde Archipelago, South Sulawesi | [146] |  |
| *Ulva lactuca* | Pari, Seribu Islands, Jakarta  Karapyak Beach, Pangandaran, West Jawa  Porok Beach, Kemadang Tanjungsari, Greweng Coasts, Jepitu, Girisubo, Gunungkidul, Yogyakarta  Karang Makassar, Mangaitan, Komodo National Park, East Nusa Tenggara  Nusa Penida Island, Bali  Pameungpeuk, Garut, West Jawa  Labuhanbua, Sumbawa Regency, West Nusa Tenggara  Cemara Beach, East Lombok, West Nusa Tenggara  Menganti Beach, Kebumen, Central Jawa  Palang Coast, Tuban, East Java | [138]  [74]  [62]  [157]  [63]  [120]  [70]; [69]  [136]  [162]  [168] |  |
| *U. rigida* | Permisan Beach Nusakambangan, Cilacap, Central Java | [117]; [71] |  |
| *U. plumosa* | Spermonde Archipelago, South Sulawesi | [146] |  |
| *U. reticulata* | Kotok Besar and Kelapa, Seribu Island, Jakarta  Pari Island, Seribu Island, Jakarta  Spermonde Archipelago, South Sulawesi  Padar, Komodo National Park, East Nusa Tenggara | [66]  [127]; [24]; [126]  [146]  [157] |  |
| *U. intestinalis* | West coast Banda Aceh, Aceh  Menganti Beach, Kebumen, Central Java | [161]  [162] |  |
| *U. rotundata* | West coast Banda Aceh, Aceh | [161] |  |
| *U. compressa* | Sepang Bay, Bengkulu | [158] |  |
| *U. fleuxosa* | Karapyak Beach, Pangandaran, West Java | [74] |  |
| *Valonia ventricosa* | Seribu Islands (Island ofTikus, Payung Kecil, Tidung Kecil, Tidung Besar, Air, Semak Daun, Kotok Besar, Kotok Kecil, Peniki, Kelapa, Panjang, Belanda, Putri, Sepa Besar, Hantu Kecil, Hantu Besar) Jakarta  Porok Beach, Kemadang Tanjungsari, Gunungkidul, Yogyakarta  Mangaitan, Komodo National Park, East Nusa Tenggara  Taka Bonerate, Selayar Island, South Sulawesi | [66]  [62]  [157]  [171] |  |
| *V. aegagropila* | Spermonde Archipelago, South Sulawesi  Padar and Mangaitan, Komodo National Park, East Nusa Tenggara  Mantehage Island, North Sulawesi | [146]  [157]  [87] |  |
| *V. macrophysa* | Spermonde Archipelago, South Sulawesi | [146] |  |
| *Valoniopsis pachynema* | West coast Banda Aceh, Aceh  Permisan Beach Nusakambangan Island, Cilacap, Central Java  Simeulue Island, Aceh  Menganti Beach, Kebumen, Central Java | [161]  [117]; [71]  [68]  [162] |  |
| *Ventricaria ventricosa* | Spermonde Archipelago, South Sulawesi | [146] |  |

Supplementary Table 2. Macroalgae from Rhodophyceae (red algae)

| Rhodophyceae | Site | References |
| --- | --- | --- |
| *Acanthopora muscoides* | Seribu Island (Island of Untung Jawa, Lancang Besar, Tidung Kecil, Tidung Besar, Air, Semak Daun , Kotok Besar, Kotok Kecil, Peniki, Kelapa, Panjang, Belanda Sepa Besar, Hantu Kecil) Jakarta  Pari Island, Seribu Island, Jakarta  Spermonde Archipelago, South Sulawesi  Padar, Komodo National Park, East Nusa Tenggara  Permisan Beach Nusakambangan Island, Cilacap  Krakal Beach, Gunungkidul, Yogyakarta | [66]  [138]  [146]  [157]  [117]; [71]  [114] |
| *A. spicifera* | Seribu Island (Island of Untung Jawa, Lancang Besar, Tidung Kecil, Tidung Besar, Air, Semak Daun , Kotok Besar, Kotok Kecil, Peniki, Kelapa, Panjang, Belanda Sepa Besar, Hantu Kecil) Jakarta  Pari Island, Seribu Island, Jakarta  Porok Beach, Gunungkidul, Yogyakarta  Spermonde Archipelago, South Sulawesi  Komodo National Park (Karang Makassar, Padar, Mangaitan, and Muntia) East Nusa Tenggara  Sepanjang Beach, Gunung kidul, Yogyakarta  Simeulue Island, Aceh  West coast Banda Aceh, Aceh  Pari Island, Seribu Island, Jakarta  Palang Coast, Tuban, East Java  Sepang Bay, Bengkulu | [66]  [138]  [62]  [146]  [157]  [132]  [68]  [161]  [163]  [168]  [158] |
| *A. dendroides* | Pari Island, Seribu Island, Jakarta  Ambon (Hutumari, Rutong, and Leahari) Maluku | [138]  [109] |
| *Actinotrichia fragilis* | Poopoh, Minahasa, North Sulawesi  Seribu Island (Island of Air, Semak Daun, Kotok Besar, Kotok Kecil, Panjang, Sepa Besar, Hantu Kecil) Jakarta  Spermonde Archipelago, South Sulawesi  Mantehage Island, North Sulawesi  Talawaan Bajo Village, Minahasa, North Sulawesi | [88]  [66]  [146]  [87]  [143] |
| *Amansia glomerata* | Spermonde Archipelago, South Sulawesi  Komodo National Park (Setuga and Padar)  Mantehage Island, North Sulawesi | [146]  [157]; [89]  [87] |
| *Amphiroa beauvoisii* | Spermonde Archipelago, South Sulawesi Olibuu, Paguyaman, Boalemo, Gorontalo | [146]  [148] |
| *A. fragilissima* | Pari Island, Seribu Island, Jakarta  Minahasa (Poopoh and Kampung Ambong) North Sulawesi  Seribu Island (Island of Air, Semak Daun, Kotok Besar, Kotok Kecil, Panjang, Sepa Besar, Hantu Kecil) Jakarta  Spermonde Archipelago, South Sulawesi  Komodo National Park (Setuga, Karang Makassar, Padar, Papagarang, Mangaitan, and Muntia) East Nusa Tenggara  Taka Bonerate, Selayar Island, South Sulawesi  Ternate Island, Maluku  Sepang Bay, Bengkulu | [163]  [88]  [66]  [146]  [157]  [171]  [164]  [158] |
| *Amphiroa spp.* | Seribu Island (Island of Bidadari, Onsrut, Untung Jawa, Bokor, Lancang Besar, Payung Kecil, Tidung Kecil, Tidung Besar, Semak Daun, Kotok Besar, Kelapa, Panjang) Jakarta | [66] |
| *A. canaliculata* | Spermonde Archipelago, South Sulawesi | [146] |
| *A. fragilissima* | Tanjung Merah, Bitung, North Sulawesi  Minahasa (Kampung Ambong, Poopoh, and Tumbak) North Sulawesi  Spermonde Archipelago, South Sulawesi  Komodo National Park (Setuga, Karang Makassar, Padar, Papagarang, Mangaitan, and Muntia) East Nusa Tenggara  Minahasa (Kora-Kora and Talawaan) North Sulawesi.  West coast Banda Aceh, Aceh | [47]; [143]  [87]; [89]  [146]  [157]  [143]  [161] |
| *A. foliacea* | Spermonde Archipelago, South Sulawesi | [146] |
| *A. rigida* | Minahasa (Kampung Ambong, Poopoh, and Tumbak) North Sulawesi  Ambon (Suli and Rutong) Maluku  Mantehage Island, North Sulawesi | [89]  [87] |
| *A. franciscana* | Krakal Beach, Gunungkidul, Yogyakarta | [114] |
| *Botryocladia microphysa* | Spermonde Archipelago, South Sulawesi | [146] |
| *Carollina Sp.* | Pari Island, Seribu Island, Jakarta | [163] |
| *Callophyllis crispata* | Menganti Beach, Kebumen, Central Java | [162] |
| *Ceratodictyon spongiosum* | Tanjung Merah, Bitung, North Sulawesi  Minahasa (Kampung Ambong, Poopoh, and Tumbak) North Sulawesi  Seribu Island (Island of Tikus, Tidung Kecil, Peniki)  Spermonde Archipelago, South Sulawesi | [74]; [143]  [89]; [87]  [66]  [146] |
| *Ceratodictyon sp.* | Seribu Island (Island of Untung Jawa, Lancang Besar, Tidung Kecil, Tidung Besar, Air, Peniki) Jakarta | [66] |
| *C. variabile* | Spermonde Archipelago, South Sulawesi | [146] |
| *C. intricatum* | Spermonde Archipelago, South Sulawesi | [146] |
| *C. repens* | Spermonde Archipelago, South Sulawesi | [146] |
| *Champia parvula* | Seribu Island (Island of Untung Jawa, Tidung Kecil, Tidung Besar, Air, Semak Daun, Kotok Besar) Jakarta  Spermonde Archipelago, South Sulawesi | [66]  [146] |
| *Chondria dasyphylla* | Spermonde Archipelago, South Sulawesi | [146] |
| *Coriophyllum setchellii* | Spermonde Archipelago, South Sulawesi | [146] |
| *Chondrus crispus* | Menganti Beach, Kebumen, Central Jawa  Palang Coast, Tuban, East Java | [162]  [168] |
| *Cryptonemia decumbens* | Spermonde Archipelago, South Sulawesi | [146] |
| *Dichotomaria marginata* | Greweng Coasts, Gunungkidul, Yogyakarta | [62] |
| *Dasya baillouviana* | Spermonde Archipelago, South Sulawesi | [146] |
| *Dasya sp.* | Seribu Island (Island of Dapur, Tikus, Payung Kecil and Jukung) | [66] |
| *D. caraibica* | Spermonde Archipelago, South Sulawesi | [146] |
| *Dermonema virens* | West coast Banda Aceh, Aceh | [161] |
| *Eucheuma spinosum* | Porok Beach, Gunungkidul, Yogyakarta  Binuangeun, Banten  Nusa Penida Island, Bali  Sumenep, Madura, East Java  Takalar, South Sulawesi  Bantaeng, South Sulawesi  Barru, South Sulawesi  Luwu, South Sulawesi  Makassar, South Sulawesi  Bulukumba, South Sulawesi  Wajo, South Sulawesi  Selayar, South Sulawesi  Ujung Genteng, Sukabumi, West Java  Labuhanbua, Sumbawa, West Nusa Tenggara  Cemara Beach, Lombok, West Nusa Tenggara  West coast Banda Aceh, Aceh  Menganti Beach, Kebumen, Central Java | [62]  [90]  [65]  [65]  [65]; [38]; [128]; [113]  [155]  [128]  [128]  [128]  [128]  [128]  [128]  [70]  [70]; [69]  [136]  [161]  [162] |
| *Eucheuma sp.* | Padar, Komodo National Park, East Nusa Tenggara | [157] |
| *E. denticulatum* | Porok and Greweng Coast, Gunungkidul, Yogyakarta  Cemara Beach, Lombok, West Nusa Tenggara  Nusa Lembongan Island, Bali  Samaringa Island, Central Sulawesi  Riau, Riau Archipelago  Takalar, South Sulawesi  Mentahage Island, South Sulawesi  Rote Island, East Nusa Tenggara  Laikang Village, Takalar, South Sulawesi  Alor Island, East Nusa Tenggara  Bulukumba, South Sulawesi  Pari Island, Seribu Island, Jakarta  Taka Bonerate, Selayar Island, South Sulawesi | [62]  [136]  [75]; [50]; [13]  [75]  [75]  [94]  [87]  [94]  [112]; [13]; [134]  [112]  [58]; [59]  [163]  [171] |
| *E. edule* | Pari Island, Seribu Island, Jakarta  Porok, Gunungkidul, Yogyakarta  Spermonde Archipelago, South Sulawesi | [75]; [138]  [62]  [146] |
| *E. serra* | Spermonde Archipelago, South Sulawesi  Permisan Beach, Nusakambangan, Cilacap, Central Java | [146]  [117]; [71] |
| *E. cottonii* | Bantaeng, South Sulawesi  Pangkep District, South Sulawesi  Pajukukang, Bantaeng, South Sulawesi | [155]  [167]  [160] |
| *E. striatus* | Serewe Village, East Lombok, West Nusa Tenggara | [156] |
| *Ellisolandia elongate* | West coast Banda Aceh, Aceh | [161] |
| *Galaxaura apiculate* | Tanjung Merah, Bitung, North Sulawesi  Mentahage Island, North Sulawesi  Talawaan Bajo Village, Minahasa, North Sulawesi | [88]  [87]  [143] |
| *Galaxaura spp.* | Seribu Island (Island of Dapur, Lancang Besar, Tikus, Payung Kecil, Tidung Besar, and Air) Jakarta  Sepang Bay, Bengkulu | [66]  [158] |
| *G. filamentosa* | Mantehage Island, North Sulawesi | [87] |
| *G. subfruticolosa* | Ambon (Humuturi, Rutong, and Laehari) Maluku  Ternate Island, Maluku | [109]  [164] |
| *G. obtusata* | Spermonde Archipelago, South Sulawesi | [146] |
| *G. striata* | Spermonde Archipelago, South Sulawesi | [146] |
| *G. rugosa* | Minahasa (Kampung Ambong, Poopoh, and Tumbak) North Sulawesi  Spermonde Archipelago, South Sulawesi  Kora-kora, Minahasa, South Sulawesi  Ujung Genteng, Minahasa, North Sulawesi  West coast Banda Aceh, Aceh  Pari Island, Seribu Island, Jakarta  Taka Bonerate, Selayar Island, South Sulawesi | [89]; [88]  [146]  [143]  [70]  [161]  [163]  [171] |
| *G. cohaerens* | Spermonde Archipelago, South Sulawesi | [146] |
| *G. kjellmanii* | Krakal Beach, Gunungkidul, Yogyakarta  Olibuu, Paguyaman, Boalemo, Gorontalo | [114]  [148] |
| *Ceramium sp.* | Spermonde Archipelago, South Sulawesi  Arungkeke waters, Jeneponto, South Sulawesi | [146]  [98] |
| *Gracilaria gigas* | Gerupuk Bay, Lombok, West Nusa Tenggara  Sekotong Beach, West Lombok, West Nusa Tenggara  Menganti Beach, Kebumen, Central Java | [64]  [162] |
| *Gracilaria eucheumoides* | Spermonde Archipelago, South Sulawesi  Ambon (Suli, Hutumuri, Rutong, and Laehari) Maluku) | [146]  [109] |
| *Gracilaria sp.* | Seribu Island (Island of Tikus, Air, Semak Daun, Kelapa, Panjang) Jakarta  Komodo National Park (Setuga, Padar and Muntia) East Nusa Tenggara.  Lontar Beach, Serang, Banten  Takalar, South Sulawesi  Arungkek Water, Jeneponto, South Sulawesi  Luwu, South Sulawesi  Tirtajaya & Cibuaya, Karawang, West Java  Domas Village, Serang, Banten  Kelapa Beach, Tuban, East Java  South Bungku, Witaponda District and Petasia District, Morowali, Central Sulawesi  Sederhana Village, Bekasi, West Java  Ujung Genteng, Sukabumi, West Java  Labuhanbua, Sumbawa, West Nusa Tenggara  South Garut, Central Java  Palang Coast, Tuban, East Java | [66]  [157]  [100]  [13]; [38]  [98]  [155]  [115]  [102]  [91]  [153]  [77]  [70]  [70]; [69]  [159]  [168] |
| *G. edulis* | Tanjung Merah, Bitung, North Sulawesi  Minahasa (Kampung Ambong, Poopoh, and Tumbak) North Sulawesi  Mantehage Island, North Sulawesi  Kora-kora adn Talawaan Bajo Village, Minahasa, South Sulawesi  Seriwe Bay, Lombok, East Nusa Tenggara | [88]  [89]; [88]  [87]  [143]  [165] |
| *G. salicornia* | Minahasa (Kampung Ambong, Poopoh, and Tumbak) North SulawesiSeribu Island (Island of Payung Kecil, Tidung Kecil, Tidung Besar, Air, Semak Daun, Peniki and Kelapa) Jakarta  Porok Beach, Gunungkidul, Yogyakarta  Spermonde Archipelago, South Sulawesi  Komodo National Park (Setuga, Mangaitan, Muntia) East Nusa Tenggara  Ambon (Suli, Hutumuri, Rutong and Laehari) Maluku  Mentehage Island, North Sulawesi  Talawaan Bajo Village, Minahasa, North Sulawesi  Nain Island, North Minahasa, North Sumatra  Ujung Genteng, Sukabumi, West Java  Labuhanbua, Sumbawa, West Nusa Tenggara  Krakal Beach, Gunungkidul, Yogyakarta  West coast Banda Aceh, Aceh  Taka Bonerate, Selayar Island, South Sulawesi  Ternate Island, Maluku  Palang Coast, Tuban, East Java  Sepang Bay, Bengkulu | [89]; [88]  [66]  [62]  [146]  [157]  [109]  [87]  [143]  [124]  [70]  [70]; [69]  [114]  [161]  [171]  [164]  [168]  [158] |
| *G. arcuata* | Porok Beach, Gunungkidul, Yogyakarta  Spermonde Archipelago, South Sulawesi  Krakal Beach, Gunungkidul, Yogyakarta  Sepang Bay, Bengkulu | [62]  [146]  [114]  [158] |
| *G. coronopifolia* | Spermonde Archipelago, South Sulawesi  West coast Banda Aceh, Aceh | [146]  [161] |
| *G. blodgettii* | Spermonde Archipelago, South Sulawesi  Ambon (Suli, Hutumuri, Rutong and Laehari) Maluku | [146]  [109] |
| *G. verrucosa* | Spermonde Archipelago, South Sulawesi  Mentahage Island, North Sulawesi  Maros, South Sulawesi  West coast Banda Aceh, Aceh  Menganti Beach, Kebumen, Central Jawa  Palang Coast, Tuban, East Java | [146]  [87]  [110]  [161]  [162]  [168] |
| *G. lichenoides* | Ambon (Suli, Hutumuri, Rutong and Laehari) Maluku  Menganti Beach, Kebumen, Central Java | [109]  [162] |
| *G. crassa* | Ambon (Suli, Hutumuri, Rutong and Laehari) Maluku  Ternate Island, Maluku | [109]  [164] |
| *G. textorii* | Tumbak, Minahasa, North Sulawesi  Mentahage Island, North Sulawesi | [88]  [87] |
| *G. foliifera* | Permisan Beach Nusakambangan Island, Cilacap. Central Java  Ujung Genteng, Sukabumi, West Jawa  Labuhanbua, Sumbawa, West Nusa Tenggara | [117]; [71]  [70]  [70]; [69] |
| *G. changii* | Takalar, Pangkep, Bone and Sinjai Regency, South Sulawesi | [154] |
| *G. gracilis* | Mentahage Island, North Sulawesi  Barru, South Sulawesi | [87]  [128] |
| *G. corticata* | West coast Banda Aceh, Aceh  Palang Coast, Tuban, East Java | [161]  [168] |
| *G. canaliculata* | Menganti Beach, Kebumen, Central Java | [162] |
| *Gelidiella acerosa* | Minahasa (Kampung Ambong, Poopoh, Tumbak) North Sulawesi  Seribu Island (Island of Bokor, Lancang Besar , Tidung Besar, Air, Semak Daun, Kelapa, Hantu Besar) Jakarta  Pari Island, Seribu Island, Jakarta  Porok Beach and Greweng Coast, Gunungkidul, Yogyakarta  Spermonde Archipelago, South Sulawesi  Komodo National Park (Setuga, Karang Makassar, Padar Kecil) East Nusa Tenggara  Ambon (Suli, Hutumuri, Rutong, Leahari) Maluku  Krakal Beach, Gunungkidul, Yogyakarta  Simeulue island, Aceh  West coast Banda Aceh, Aceh | [89]  [66]  [138]  [62]  [146]  [157]  [109]  [114]  [68]  [161] |
| *Gelidium pusillum* | Spermonde Archipelago, South Sulawesi | [146] |
| *Gigartina stellata* | Menganti Beach, Kebumen, Central Java | [162] |
| *Grateloupia sp.* | Spermonde Archipelago, South Sulawesi | [146] |
| *Griffithsia sp.* | Spermonde Archipelago, South Sulawesi | [146] |
| *Gymnogongrus sp.* | Spermonde Archipelago, South Sulawesi | [146] |
| *Halymenia sp.* | Libukang Island, Jeneponto, South Sulawesi  Binuangeun, Banten | [130]  [90]; [140] |
| *Halymenia floresia* | Spermonde Archipelago, South Sulawesi | [146] |
| *H. durvillaei* | Seribu Island (Untung Jawa, Lancang Besar and Payung Kecil Island) Jakarta  Spermonde Archipelago, South Sulawesi  Kayangan Island, Ujung Pandang, South Sulawesi | [66]  [146]  [86] |
| *H. acuminata* | Spermonde Archipelago, South Sulawesi | [146] |
| *H. dilatata* | Seribu Island (Lancang Besar, Payung Kecil and Air Island) Jakarta  Spermonde Archipelago, South Sulawesi | [66]  [146] |
| *H. amoena* | Spermonde Archipelago, South Sulawesi | [146] |
| *H. tondoana* | Seribu Island (Untung Jawa, Lancang, Besar, Payung Kecil) Jakarta | [66] |
| *H. maculeata* | Seribu Island (Lancang, Besar, Payung Kecil, Air) Jakarta | [66] |
| *H. malaysiana* | Seribu Island (Lancang, Besar, Payung Kecil, Air) Jakarta | [66] |
| *H. harveyana* | Menganti Beach, Kebumen, Central Java | [162] |
| *Hydrolithon gardineri* | Spermonde Archipelago, South Sulawesi | [146] |
| *Hydrolithon onkodes* | Spermonde Archipelago, South Sulawesi | [146] |
| *Hydrolithon reinboldii* | Spermonde Archipelago, South Sulawesi | [146] |
| *Hypnea pannosa* | Spermonde Archipelago, South Sulawesi; Krakal Beach, Gunungkidul, Yogyakarta | [146]  [114]; [80] |
| *Hypena sp.* | Seribu Island (Island of Ayer Besar, Damar Kecil, Lancang Besar, Tikus, Tidung Kecil, Tidung Besar, Air, Kotok Besar, Kotok Kecil, Peniki, Panjang, Putri) Jakarta  Muntia, Komodo National Park, East Nusa Tenggara | [66]  [157] |
| *Hypnea asperi* | Komodo National Park (Padar Kecil, Padar and Mangaitan) East Nusa Tenggara  Pari Island, Seribu Island, Jakarta  Taka Bonerate, Selayar Island, South Sulawesi | [157]  [163]  [171] |
| *H. musciformis* | Spermonde Archipelago, South Sulawesi  Arungkek Waters, Jeneponto, South Sulawesi  West coast Banda Aceh, Aceh | [146]  [98]  [161] |
| *H. spinella* | Spermonde Archipelago, South Sulawesi  Mantehage Island, North Sulawesi | [146]  [87] |
| *H. sevicornis* | Ambon (Suli, Hutumuri, Rutong, Laehari) Maluku  Ternate Island, Maluku | [109]  [164] |
| *H. charoides* | Spermonde Archipelago, South Sulawesi | [146] |
| *H. cornuta* | West coast Banda Aceh, Aceh | [161] |
| *H. pannosa* | West coast Banda Aceh, Aceh  Spermonde Archipelago, South Sulawesi  Krakal Beach, Gunungkidul, Yogyakarta | [161]  [146]  [114] |
| *H. boergesenii* | Tanjung Merah, Bitung, North Sulawesi  Minahasa (Kampong Ambong, Poopoh, Tumbak) North Sulawesi  Spermonde Archipelago, South Sulawesi  Kora-kora, Minahasa, North Sulawesi  Talawaan Bajo Village, Minahasa, North Sulawesi | [89]  [89]; [88]  [146]  [89]  [89] |
| *Herposiphonia secunda* | Spermonde Archipelago, South Sulawesi | [146] |
| *Jania arberescus* | Ambon (Suli, Hutumuri, Rutong) Maluku | [109] |
| *J. rubens* | Ambon (Suli, Hutumuri, Rutong, Laehari) Maluku | [109] |
| *J. Intermedia* | Simeulue Island, Aceh | [68] |
| *J. adherens* | Olibuu, Paguyaman, Boalemo, Gorontalo  West coast Banda Aceh, Aceh | [148]  [161] |
| *Kallymenia feldmannii* | Spermonde Archipelago, South Sulawesi | [146] |
| *Kappaphycus alvarezii* | Minahasa (Kampong Ambong, Poopoh, Tumbak) North Sulawesi  Pari Island, Seribu Island, Jakarta  Porok Beach and Greweng Coast, Gunungkidul, Yogyakarta  Spermonde Archipelago, South Sulawesi  Nusa Lembongan Island, Bali  Salakan and Donggala, Central Sulawesi  Takalar, South Sulawesi  Talango Island, Sumenep, East Java  Arungkeke Water, Jeneponto, South Sulawesi  North Laut and East Laut Island, Kotabaru, South Kalimantan  Karimunjawa Island, Central Jawa  Bungin Permai, South Konawe, Southeast Sulawesi  Purirano, Kendari, Southeast Sulawesi  South Lampung, Lampung  Serang, Banten  East Lombok, West Nusa Tenggara  Kupang, East Nusa Tenggara  Pangkep, South Sulawesi  Bantaeng, South Sulawesi  Mamuju, West Sulawesi  North Gorontalo, Gorontalo  North Minahasa, North Sulawesi  Bontang, East Kalimantan  Maros, South Sulawesi  Lemo, Bombana, Southeast Sulawesi  Rote Island, East Nusa Tengara  Gerupuk Bay, Lombok, East Nusa Tenggara  Barru, South Sulawesi  Laikang Village, Takalar, South Sulawesi  Luwu, South Sulawesi  Lembo and Wawolesea, North Konawe, Southeast Sulawesi  Serewe Village, Lombok, West Nusa Tenggara  Nunukan Island, Nunukan Regency, North Kalimantan  Kastele, Ternate Island, North Maluku  East Luwu, South Sulawesi  Makassar, South Sulawesi  District Kasimbar and Mepanga, Parigi Moutong, Central Sulawesi  Tesabela Waters, Kupang, East Nusa Tenggara  Muna, Southeast Sulawesi  Bulukumba, South Sulawesi  Indari Waters, South Halmahera, North Maluku  Lakeba, Southeast Sulawesi  Amal and Tanjang Pasir, Tarakan, East Kalimantan  Kelapa Beach, Tuban, East Java  South Bungku, District Witaponda and Petasia, Morowali, Central Sulawesi  Sulamu Waters, Kupang, East Nusa Tenggara  West Sorkam, Tapanuli, North Sumatera  Lobo Hede Village, Sawu Island, East Nusa Tenggara  Wajo and Selayar, South Sulawesi  Mandalle Beach, Pangkep, South Sulawesi  Salemo Island, Pangkep, South Sulawesi  Mandar Bay, Majene, West Sulawesi  Posi-posi Waters, South Halmahera, North Maluku  Batubao Waters, Kupang, East Nusa Tenggara  Pahunga Ludo, East Sumba, East Nusa Tenggara  Liang Village, Banggai, Central Sulawesi  Panjang Island, Serang District, Banten | [89]; [88]  [24]; [126]; [57]; [99]; [97]  [62]  [146]  [116]; [50]; [13]; [155]  [147]; [13]; [38]; [141]; [103]; [128]  [61]  [98] ; [141]  [145]  [142]; [151]; [57]  [52]; [119]; [54]  [52]  [133]  [133]  [133]  [133]  [133]; [103]  [133]; [141]  [133]; [111]  [133]; [112]  [133]  [133]; [60]  [141]  [52]; [54]; [101]  [96]  [69]; [137]  [128]  [112]; [156]; [13]  [155]; [128]  [101]  [156]; [79]  [142]; [48]  [46]  [141]  [141]; [128]; [122]  [92]  [107]; [149]  [101]  [48]; [58]; [128]  [78]  [85]  [55]  [91]  [153]  [106]  [93]  [112]  [128]  [121]  [121]  [76]  [78]  [108]  [135]  [125]  [166]  [170] |
| *K. striatum* | Spermonde Archipelago; South Sulawesi  West coast Banda Aceh, Aceh | [146]  [161] |
| *Laurencia papillosa* | Tanjung Merah, Bitung, North Sulawesi  Minahasa (Kampung Ambong, Poopoh, Tumbak) North Sulawesi  Spermonde Archipelago, South Sulawesi  Mantehage Island, North Sulawesi  Talawaan Bajo Village, Minahasa, North Sulawesi | [74]  [89]; [88]  [146]  [87]  [143] |
| *L. nidifica* | Pari Island, Seribu Island, Jakarta | [163] |
| *Laurencia sp.* | Seribu Island (Island of Ayer Besar, Damar Kecil, Untung Jawa, Bokor, Lancang Besar, Payung Kecil, Tidung Kecil, Tidung Besar, Air, Semak Daun, Kotok Kecil, Peniki, Belanda and Putri) Jakarta  Pari Island, Seribu Island, Jakarta  Simeulue Island, Aceh | [66]  [138]  [68] |
| *L. doty* | Spermonde Archipelago, South Sulawesi | [146] |
| *L. pinnata* | Spermonde Archipelago, South Sulawesi | [146] |
| *L. brongniartii* | Spermonde Archipelago, South Sulawesi | [146] |
| *L. parvipapillata* | Spermonde Archipelago, South Sulawesi | [146] |
| *L. mariannensis* | Spermonde Archipelago, South Sulawesi | [146] |
| *L. obtusa* | Spermonde Archipelago, South Sulawesi  Padar and Mangaitan, Komodo National Park, East Nusa Tenggara | [146]  [157] |
| *L. cartilaginea* | Spermonde Archipelago, South Sulawesi | [146] |
| *L. glandulifera* | Spermonde Archipelago, South Sulawesi | [146] |
| *Leveillea jungermannioides* | Spermonde Archipelago, South Sulawesi | [146] |
| *Leptofauchea sp.* | Porok Beach, Gunungkidul, Yogyakarta | [62] |
| *Lithophvllum bamleri* | Spermonde Archipelago, South Sulawesi | [146] |
| *L. kotschyanum* | Spermonde Archipelago, South Sulawesi | [146] |
| *L. okamurai* | Spermonde Archipelago, South Sulawesi | [146] |
| *L. tamiense* | Spermonde Archipelago, South Sulawesi | [146] |
| *Lithothamnionprolifer* | Spermonde Archipelago, South Sulawesi | [146] |
| *Liagora caenomyce* | Ambon (Suli, Hutumuri, Rutong and Leahari) Maluku | [109] |
| *Liagora sp.* | Seribu Island (Island of, Lancang Besar, Tikus, Payung Kecil, Tidung Besar) | [66] |
| *L. ceranoides* | Spermonde Archipelago, South Sulawesi  Suli and Rutong, Ambon, Maluku | [146]  [109] |
| *Mastophora pacifica* | Spermonde Archipelago, South Sulawesi | [146] |
| *M. rosea* | Minahasa (Kampung Ambong, Poopoh and Tumbak) North Sulawesi  Spermonde Archipelago, South Sulawesi  Mantehage Island, North Sulawesi | [89]  [146]  [87] |
| *Meristotheca papulosa* | Spermonde Archipelago, South Sulawesi | [146] |
| *Mesophyllum erubescens* | Spermonde Archipelago, South Sulawesi | [146] |
| *M. syrphetodes* | Spermonde Archipelago, South Sulawesi | [146] |
| *Neogoniolithon fosliei* | Spermonde Archipelago, South Sulawesi | [146] |
| *Neosiphonia apiculate* | Arungkeke, Jenponto, South Sulawesi | [98] |
| *Peyssonnelia squamaria* | Spermonde Archipelago, South Sulawesi | [146] |
| *Polysiphonia infestans* | Spermonde Archipelago, South Sulawesi | [146] |
| *Portieria hornemannii* | Greweng Coasts, Gunungkidul, Yogyakarta  Spermonde Archipelago, South Sulawesi  Mangaitan, Komodo National Park, East Nusa Tenggara  Menganti Beach, Kebumen, Central Java | [62]  [146]  [157]  [162] |
| *Portieria sp.* | Seribu Island (Island of Damar Besar, Untung Jawa, Dapur, Lancang Besar, Tikus, Tidung Kecil, Tidung Besar, Semak Daun, Belanda, Jukung, Hantu Kecil) Jakarta. | [66] |
| *Pterocladia caerulescens* | Damar Kecil Island, Seribu Island, Jakarta  Spermonde Archipelago, South Sulawesi | [66]  [146] |
| *Pterocholla capillacea* | West coast Banda Aceh, Aceh | [161] |
| *P. caloglossoides* | Spermonde Archipelago, South Sulawesi | [146] |
| *Rhodymenia pacifica* | Spermonde Archipelago, South Sulawesi | [146] |
| *R. palmata* | Menganti Beach, Kebumen, Central Java | [162] |
| *Rhodymenia sp.* | Krakal Beach, Gunungkidul, Yogyakarta | [114] |
| *R. coacta* | Spermonde Archipelago, South Sulawesi | [146] |
| *R. carollina* | Spermonde Archipelago, South Sulawesi | [146] |
| *R. intricata* | Spermonde Archipelago, South Sulawesi | [146] |
| *R. leptophyllia* | Spermonde Archipelago, South Sulawesi | [146] |
| *R. palmata* | Permisan Beach Nusakambangan Island, Cilacap, Central Java | [117]; [71] |
| *Sporolithon episoredion* | Spermonde Archipelago, South Sulawesi | [146] |
| *S. episporum* | Spermonde Archipelago, South Sulawesi | [146] |
| *S. molle* | Spermonde Archipelago, South Sulawesi | [146] |
| *S. ptychoides* | Spermonde Archipelago, South Sulawesi | [146] |
| *Spyridia filamentosa* | Seribu Islands (Island of Payung Kecil, Tidung Kecil, Tidung Besar, Air, Semak Daun, Kotok Kecil, Panjang, Putri, Sepa Besar) Jakarta  Spermonde Archipelago, South Sulawesi Mantehage Island, North Sulawesi | [66]  [146]  [87] |
| *S. fusiformis* | Simeulue Island, Aceh | [68] |
| *Titanophora weberae* | Spermonde Archipelago, South Sulawesi | [146] |
| *Titanophora sp.* | Tikus and Payung Kecil Island, Seribu Island, Jakarta  Krakal Beach, Gunungkidul, Yogyakarta | [66]  [114] |
| *Tricleocarpa fragilis* | Ambon (Suli, Hutumuri, Rutong and Leahari) Maluku | [109] |
| *Trichogloea requienii* | Spermonde Archipelago, South Sulawesi | [146] |
| *Zellera tawallina* | Tidung Kecil and Tidung Besar, Seribu Islands, Jakarta  Spermonde Archipelago, South Sulawesi | [66]  [146] |

Supplementary Table 3. Macroalgae from Phaeophyceae (brown algae)

| Phaeophyceae | Site | Reference |
| --- | --- | --- |
| *Colpomenia* | Libukang Island, Jeneponto, South Sulawesi | [130] |
| *Colpomenia sinuosa* | Seribu Islands (Island of Damar Besar, Untung Jawa, Bokor, Tidung Kecil, Kotok Besar, Kotok Kecil, Peniki, Kelapa, Panjang, Belanda, Putri, Hantu Kecil) Jakarta | [66] |
| *Dictyopteris repens* | Spermonde Archipelago, South Sulawesi | [146] |
| *D. jamaicensis* | Spermonde Archipelago, South Sulawesi | [146] |
| *Dictyota bartayresiana* | Spermonde Archipelago, South Sulawesi  Ujung Genteng, Sukabumi, West Java  Olibuu, Paguyaman, Boalemo, Gorontalo  Pari Island, Seribu Island, Jakarta  Taka Bonerate, Selayar Island, South Sulawesi | [146]  [69]  [148]  [163]  [171] |
| *D. ciliolata* | Spermonde Archipelago, South Sulawesi | [146] |
| *D. ceylanica* | West Coast, Aceh | [161] |
| *D. fasciola* | West Coast, Aceh | [161] |
| *D. cervicornis* | Spermonde Archipelago, South Sulawesi  Olibuu, Paguyaman, Boalemo, Gorontalo | [146]  [148] |
| *D. Intermedia* | Spermonde Archipelago, South Sulawesi | [146] |
| *D. marginata* | Spermonde Archipelago, South Sulawesi | [146] |
| *D. dichotoma* | Tanjung Merah, Bitung, North Sulawesi  Kampung Ambong, Poopoh, and Tumbak, Minahasa, North Sulawesi  Spermonde Archipelago, South Sulawesi  Komodo National Park (Karang Makassar, Papagarang and Muntia)  Mentahage Island, North Sulawesi  Kora-kora and Talawaan, Minahasa, North Sulawesi  Ujung Genteng, Sukabumi, West Java  Labuhanbua, Sumbawa, West Nusa Tenggara  Prigi Bay, Trenggalek, East Java  Menganti Beach, Kebumen, Central Java | [47]; [143]  [89]; [88]  [146]  [157]  [87]  [143]  [70]  [70]; [69]  [84]  [150] |
| *D. patens* | Ambon (Suli, Hutumuri, Rutong and Laehari) Maluku | [109] |
| *Hormophysa cuneiformis* | Seribu Islands (Island of Bokor, Tidung Kecil, Tidung Besar, Air, Semak Daun, Kotok Besar, Kelapa, Panjang, Belanda, Hantu Besar) Jakarta  Spermonde Archipelago, South Sulawesi  Muntia, Komodo National Park, East Nusa Tenggara  Cemara Beach, Lombok, West Nusa Tenggara | [66]  [146]  [157]  [89] |
| *Hormophysa sp.* | Along the Bitung-Bentena Coast, North Sumatra | [152] |
| *H. triquestra* | Olibuu, Paguyaman, Boalemo, Gorontalo | [148] |
| *Hydroclathrus clathratus* | Minahasa (Kampung Ambong, Poopoh, Tumbak) North Sulawesi  Seribu Islands (Island of Payung Kecil, Tidung Besar, Semak Daun, Kotok Besar, Kotok Kecil, Panjang, Belanda, Putri, Sepa Besar) Jakarta  Spermonde Archipelago, South Sulawesi  Ambon (Suli, Hutumuri, Rutong and Laehari) Maluku  Setuga, Komodo National Park, East Nusa Tenggara  Mantehage Island, North Sulawesi  Labuhanbua, Sumbawa, West Nusa Tenggara | [89]; [88]  [66]  [146]  [109]  [157]  [87]; [70]  [69] |
| *H. tenuis* | Spermonde Archipelago, South Sulawesi | [146] |
| *Padina australis* | Minahasa (Kampung Ambong, Poopoh, Tumbak) North Sulawesi  Pari Island, Seribu Island, Jakarta  Porok Beach, Gunungkidul, Yograkarta  Spermonde Archipelago, South Sulawesi  Komodo National Park (Setugas, Mangaitan, Muntia) East Nusa Tenggara  Ambon (Suli, Hutumuri, Rutong and Laehari) Maluku  Mantehage, North Sulawesi  Talawaan Bajo Village, Minahasa, North Sulawesi  Karimunjawa Island, Central Java  Awur Bay, Jepara, Central Java  Kelapa Beach, Tuban, East Java  Lhok Bubon, West Aceh, Aceh  Prigi Bay, Trenggalek, East Java  Menganti Beach, Kebumen, Central Java  Cemara Beach, Lombok, West Nusa Tenggara  Olibuu, Paguyaman, Boalemo, Gorontalo  Taka Bonerate, Selayar Island, South Sulawesi  Palang Coast, Tuban, East Java  Permisan Beach Nusakambangan, Central Java | [89]; [88]  [127]; [24]; [126]; [138]  [62]  [146]  [157]  [109]  [87]  [143]  [118]  [114]  [91]  [72]  [84]  [150]  [136]  [148]  [171]  [168]  [117]; [71] |
| *Padina sp* | Along the Bitung-Bentena Coast, North Sulawesi  Seribu Islands (Island of Bidadari, Kelor, Ayer Besar, Damar Besar, damar Kecil, Untung Jawa, Bokor, Dapur, Lancang Besar, Tikus, Payung Kecil, Tidung Kecil, Tidung Besar, Air, Semak Daun, Kotok Besar Kotok Kecil, Peniki, Kelapa, Panjang, Belanda, Putri, Sepa Besar, Jukung, Hantu Kecil, Hanti Besar) Jakarta  Sepanjang Beach, Gunungkidul, Yogyakarta  Awur Bay, Jepara, Central Jawa  Labuhanbua, Sumbawa, West Nusa Tenggara | [152]  [66]  [132]  [70]  [69] |
| *P. boergesenii* | Spermonde Archipelago, South Sulawesi | [146] |
| *P. sanctae-crucis* | Spermonde Archipelago, South Sulawesi | [146] |
| *P. crassa* | Ambon (Suli, Hutumuri, Rutong and Laehari) Maluku  Bandengan Beach, Jepara, Central Java | [109]  [49] |
| *P. minor* | Kampung Ambong, Poopoh, Tumbak, Minahasa, North Sumatra  Pramuka Island, Seribu Island, Jakarta  Spermonde Archipelago, South Sulawesi  Ambon (Suli, Hutumuri, Rutong and Laehari) Maluku  Kora-kora, Minahasa, North Sulawesi  Simeulue Island, Aceh  Sepang Bay, Bengkulu  West Coast, Aceh | [89]  [105]  [146]  [109]  [143]  [68]  [158]  [161] |
| *P. tetrastromatica* | Pari Island, Seribu Island, Jakarta | [163] |
| *P. australis* | Pari Island, Seribu Island, Jakarta  Menganti Beach, Kebumen, Central Java  West Coast, Aceh | [163]  [162]  [161] |
| *P. antillarum* | West Coast, Aceh | [161] |
| *Rosenvingea fastigiata* | Payung Kecil dan Sepa Besar, Seribu Island, Jakarta | [66] |
| *Rosenvingea intricata* | Payung Kecil and Tidung Besar, Seribu Island, Jakarta  Spermonde Archipelago, South Sulawesi | [66]  [146] |
| *Stypopodium flabelliforme* | Air and Semak Daun Island, Seribu Island, Jakarta  Spermonde Archipelago, South Sulawesi | [66]  [146] |
| *Stypopodium zonale* | Spermonde Archipelago, South Sulawesi | [146] |
| *Sargassum cristaefolium* | Porok Beach, Greweng Coast, Gunungkidul, Yogyakarta  Spermonde Archipelago, South Sulawesi  Mentahage, North Sumatra | [62]  [146]  [87] |
| *Sargassum sp.* | Setuga, Muntia, Komodo National Park, East Nusa Tenggara  Salakan, Donggala, Central Sulawesi  Karimunjawa Island, Central Jawa  Awur Bay, Jepara, Central Jawa  Permisan Beach Nusakambangan Island, Cilacap, Central Jawa  Labuhanbua, Sumbawa, West Nusa Tenggara  Pari Island, Seribu Island, Jakarta | [157]  [135]  [118]  [132]  [117]; [71]  [70]; [69]  [162] |
| *S. polycystum* | Pari Island, Seribu Island, Jakarta  Porok Beach, Greweng Coast, Gunungkidul, Yogyakarta  Muntia, Komodo Nasioal Park  Permisan Beach, Nusakambangan, Cilacap, Central Java  Awur Bay, Jepara, Central Jawa  Menganti Beach, Kebumen, Central Java  Olibuu, Paguyaman, Boalemo, Gorontalo  West Coast, Aceh | [127; [24]; [126]  [62]  [157]  [117] ; [71]  [114]  [150]  [148]  [161] |
| *S. aquifolium* | Ekas Bay, Lombok, West Nusa Tenggara | [131] |
| *S. crassifolium* | Along the Bitung-Bentena Coast, North Sulawesi  Pari Island, Seribu Island, Jakarta  Porok Beach, Kemedang, Gunungkidul, Yogyakarta  Ambon (Suli, Hutumuri, Rutong, Leahari) Maluku  Kora-kora, Minahasa, North Sulawesi  Awur Bay, Jepara, Central Java  Batu Layar, West Lombok, West Nusa Tenggara  Menganti Beach, Kebumen, Central Java  Sepang Bay, Bengkulu  West Coast, Aceh | [152]  [138]  [62]  [109]  [143]  [132]  [99]  [150]; [162]  [158]  [161] |
| *S. duplicatum* | Ambon (Suli, Hutumuri, Rutong and Laehari) Maluku  Talango Island, Sumenep, East Java  Bandengan Beach, Jepara, Central Java  Permisan Beach Nusakambangan Island, Cilacap, Central Jawa  Menganti Beach, Kebumen, Central Java  Cemara Beach, East Lombok, West Nusa Tenggara  Olibuu, Paguyaman, Boalemo, Gorontalo  West Coast, Aceh | [109]  [61]  [49]  [117]; [71]  [150]  [89]  [148]  [161] |
| *S. vulgare* | Greweng Coast, Gunungkidul, Yogyakarta  West Coast, Aceh | [62]  [161] |
| *S. crispifolium* | Ambon (Suli, Hutumuri, Rutong and Laehari) Maluku | [109] |
| *S. hawaiiesnsis* | Spermonde Archipelago, South Sulawesi | [146] |
| *S. ilicifolium* | Spermonde Archipelago, South Sulawesi  Bandengan Beach, Jepara, Central Jawa  Lhok Bubon, West Aceh, Banda Aceh  West Coast, Aceh | [146]  [139]  [72]  [161] |
| *S. pallidum* | Spermonde Archipelago, South Sulawesi | [146] |
| *S. siliquosum* | Spermonde Archipelago, South Sulawesi | [146] |
| *S. hemiphyllum* | Spermonde Archipelago, South Sulawesi | [146] |
| *S. binderi* | West Coast, Aceh | [161] |
| *S. fluitan* | West Coast, Aceh | [161] |
| *S. granuliferum* | West Coast, Aceh | [161] |
| *S. lineariforium* | West Coast, Aceh | [161] |
| *S. muticum* | West Coast, Aceh | [161] |
| *Turbinaria decurrens* | Libukang Island, Jeneponto, South Sulawesi  Ekas Bay, Lombok, West Nusa Tenggara  Kampung Ambong, Poopoh, Tumbak, Minahasa, North Sulawesi  Spermonde Archipelago, South Sulawesi  Panjang Island, Jepara, Central Jawa  West Coast, Aceh | [130]  [131]  [89]  [146]  [132]  [161] |
| *Turbinaria sp* | Seribu Islands (Island of Damar Kecil, Damar Besar, Untung Jawa, Bokor, Lancang Besar, Tikus, Payung Kecil, Tidung Kecil, Tidung Besar, Air, Semaik Daun, Kotok Besar, Kotok Kecil, Peniki, Kelapa, Belanda, Putri, Sepa Besar, Jukung, Hantu Kecil, Hantu Besar) Jakarta  Labuhanbua, Sumbawa, West Nusa Tenggara | [66]  [70]; [69] |
| *T. ornata* | Kampung Ambong, Poopoh, Tumbak, Minahasa, North Sumatera  Porok Beach, Gunungkidul, Yogyakarta  Spermonde Archipelago, South Sulawesi  Mangaitan, Komodo National Park, East Nusa Tenggara  Ambon (Suli, Hutumuri, Rutong and Laehari) Maluku  Kora-kora and Talawaan, Minahasa, North Sulawesi  Krakal Beach, Gunungkidul, Yogyakarta  Simeulue Island, Aceh  Cemara Beach, Lombok, West Nusa Tenggara  Olibuu, Paguyaman, Boalemo, Gorontalo  West Coast, Aceh | [89]  [62]  [146]  [157]  [109]  [143]  [114]  [68]  [89]  [148]  [161] |
| *T. parvifolia* | Spermonde Archipelago, South Sulawesi | [146] |
| *T. conoides* | Libukang Island, Jeneponto, South Sulawesi  Along the Bitung-Bentena Coast, North Sulawesi  Ekas Bay, Lombok, West Nusa Tenggara  Pari Island, Seribu Island, Jakarta  Spermonde Archipelago, South Sulawesi  Komodo National Park (Setuga, Padar Kecil, Mangaitan and Muntia) East Nusa Tenggara | [130]  [152]  [131]  [127]; [105]; [24]; [126]  [146]  [157] |
